# Apparent Genetic Rescue of Adult *Shank3* Exon 21 Insertion Mutation Mice Tempered by Appropriate Control Experiments

**DOI:** 10.1523/ENEURO.0317-19.2019

**Published:** 2019-09-20

**Authors:** Haley E. Speed, Mehreen Kouser, Zhong Xuan, Shunan Liu, Anne Duong, Craig M. Powell

**Affiliations:** 1 Department of Neurobiology, University of Alabama at Birmingham School of Medicine, Birmingham 35294-2182, AL; 2 Civitan International Research Center at UAB, Birmingham 35294-2182, AL

**Keywords:** autism, Cre-recombinase, reversal, *Shank3*, tamoxifen

## Abstract

*SHANK3* (*ProSAP2*) is among the most common genes mutated in autism spectrum disorders (ASD) and is the causative gene in Phelan–McDermid syndrome (PMS). We performed genetic rescue of *Shank3* mutant phenotypes in adult mice expressing a *Shank3* exon 21 insertion mutation (*Shank3^G^*). We used a tamoxifen-inducible Cre/loxP system (*Cre^Tam^*) to revert *Shank3^G^* to wild-type (WT) *Shank3^+/+^*. We found that tamoxifen treatment in adult *Shank3^G^Cre^Tam^+* mice resulted in complete rescue of SHANK3 protein expression in the brain and appeared to rescue synaptic transmission and some behavioral differences compared to *Shank3^+/+^Cre^Tam^+* controls. However, follow-up comparisons between vehicle-treated, WT Cre-negative mice (*Shank3^+/+^Cre^Tam^−* and *Shank3^+/+^Cre^Tam^+*) demonstrated clear effects of *Cre^Tam^* on baseline synaptic transmission and some behaviors, making apparently positive genetic reversal effects difficult to interpret. Thus, while the *Cre^Tam^* tamoxifen-inducible system is a powerful tool that successfully rescues *Shank3* expression in our *Shank3^G/G^* reversible mutants, one must exercise caution and use appropriate control comparisons to ensure sound interpretation.

## Significance Statement

Temporally and spatially controlled genetic reversal of mouse models of autism are used to determine critical windows in development for successful treatment. This study provides a clear example that any attempt at genetic reversal must be accompanied by all appropriate controls, including expression of the *Cre^Tam^* transgene in wild-type (WT) animals, for accurate interpretation of the genetic rescue result. In addition, this study provides two additional independent replications of behavioral and synaptic electrophysiologal abnormalities in *Shank3* exon 21 mutant mouse models in the *Cre^Tam^*-negative cohorts. Reproducibility and rigor are important and sometimes overlooked aspects of many mutant mouse behavioral and electrophysiological studies.

## Introduction

*SHANK3* (*ProSAP2*) is one of the most common genes associated with ASD and is implicated in bipolar disorder, schizophrenia, and Alzheimer’s disease (for review, see Grabrucker et al., 2011; Guilmatre et al., 2014). The *SHANK3* gene is located on the long arm of chromosome 22 at position 22q13.3 and is the causative gene in Phelan–McDermid syndrome (PMS; Bonaglia et al., 2001, 2006; Dhar et al., 2010; Boccuto et al., 2013). The gene encodes SHANK3, a postsynaptic scaffolding protein that interacts directly or indirectly with AMPA receptors (Uchino et al., 2006; Raynaud et al., 2013), NMDA receptors (Naisbitt et al., 1999), metabotropic glutamate receptors (Verpelli et al., 2011), and the actin cytoskeleton (Lim et al., 1999; Naisbitt et al., 1999; Sheng and Kim, 2000; Böckers et al., 2001) at excitatory synapses in the brain.

Human *SHANK3* mutations and changes in copy number have been modeled in mouse models (for review, see Jiang and Ehlers, 2013). Our laboratory has characterized four independent mouse lines by targeting exons 4–9 (Jaramillo et al., 2016), exon 13 (Jaramillo et al., 2017), and exon 21 (Kouser et al., 2013; Speed et al., 2015) of *Shank3*.

We have reported consistent biochemical, behavioral, and physiologic findings in two *Shank3* mouse models targeting the C-terminal domain of the SHANK3 protein (exon 21). One of these models was made by deletion of exon 21 (*Shank3^ΔC^*), loosely mimicking an autism-associated, human guanine insertion mutation that caused a frameshift mutation and premature STOP codon in exon 21 (Kouser et al., 2013). We identified behavioral deficits in *Shank3^ΔC/ΔC^* mice, including novelty avoidance and motor coordination abnormalities (Kouser et al., 2013). Synaptic transmission and synaptic plasticity were also decreased in *Shank3^ΔC/ΔC^* mice in area CA1 of the hippocampus (Kouser et al., 2013).

More recently, we have mimicked this autism-associated *SHANK3* mutation by inserting a guanine nucleotide at position 3728 of *Shank3* to cause an equivalent frameshift mutation and premature STOP codon in exon 21 (Speed et al., 2015). This *Shank3^G^* mouse shared similar phenotypes with the *Shank3^ΔC^* mouse, including loss of SHANK3 protein isoforms, novelty avoidance, motor deficits, and deficits in synaptic transmission in area CA1 of the hippocampus (Speed et al., 2015). We engineered the *Shank3^G^* mutant model as a Cre-recombinase-dependent, genetically reversible model (Speed et al., 2015). In our original publication, we demonstrated that genetic reversal of the *Shank3^G^* mutation by Cre-recombinase restored SHANK3 protein to levels indistinguishable from wild-type (WT) *Shank3^+/+^* (Speed et al., 2015). The common phenotypes of *Shank3^G^* and *Shank3^ΔC^* mouse lines underscored the robust, reproducible nature of these findings for future studies.

In the present study, we sought to answer whether autism caused by *SHANK3* mutation is a “hard-wired,” irreversible neurodevelopmental disorder or a disorder of brain function that can be reversed by normalizing *SHANK3* expression following completion of brain development. This question has important ramifications for both targeting and timing of potential therapeutic strategies. We hypothesized that adult-induced genetic reversal of *Shank3^G^* mutant mice would result in rescue of behavioral and electrophysiologic abnormalities in our genetically reversible *Shank3^G^* mutant model. This hypothesis has was examined using a similar genetic reversal strategy in other autism-related mouse models including *MeCP2* (Guy et al., 2007), *Ube3a* (Silva-Santos et al., 2015), *Syngap1* (Clement et al., 2012), and *Shank3* (Mei et al., 2016) mutants.

Our findings in Cre-negative mice replicated our previously published behavioral and synaptic abnormalities in the *Shank3^G^* mouse line, further underscoring the robust and reproducible nature of these findings. At first glance, our results in tamoxifen-treated, Cre-positive, genetically reversed mice appeared to demonstrate rescue of some, but not all, phenotypes. Our test of this hypothesis, however, included critical controls run in parallel with the key genetic reversal experiments that illuminated potential caveats to interpretation of genetic reversal experiments using the *Cre^Tam^* transgenic mouse line (Hayashi and McMahon, 2002; Guy et al., 2007; Clement et al., 2012; Silva-Santos et al., 2015; Kool et al., 2016; Mei et al., 2016).

## Materials and Methods

### Generation and genotyping of *Shank3^G/G^* mice with and without *Cre^Tam^*

Construction of the genetically reversible, *Shank3* exon 21 insertion mutant targeting vector and resulting genetically reversible *Shank3^G/G^* mouse line were described previously (Speed et al., 2015). Genotyping for *Shank3* was performed as described (Speed et al., 2015) with two primers: 21M-loxP1-sequence-sense (CTGTTGGTGTCAGTTCTTGCAGATG, in intron 20) and 21M-sequence-loxP2-antisense (CAAGGATGCTGGCCATTGAATGGCTTC, in exon 21). PCR products for WT *Shank3* and *Shank3^G^* alleles were 596 and 638 bp, respectively. Following Cre-recombination, the PCR product of the recombined *Shank3^G-Rev^* allele was 680 bp. Genotyping for the *Cre^Tam^* transgene was performed with two PCR primers: sense (GCGGTCTGGCAGTAAAAACTATC) and antisense (GTGAAACAGCATTGCTGTCACTT). PCR product for *Cre^Tam^*transgene gene was ∼100 bp.

**Figure 1.**
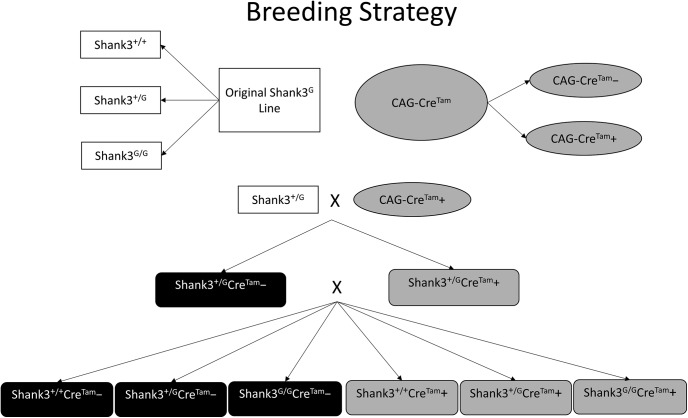
Breeding strategy for generating the *Shank3^G^Cre^Tam^* mouse line. Heterozygous *Shank3^+/G^* mice from the original *Shank3^G^* mouse line were crossed with a tamoxifen-inducible *Cre^Tam^* transgenic mouse line to produce *Shank3^+/G^Cre^Tam^−* and *Shank3^+/G^Cre^Tam^+* offspring. *Shank3^+/G^Cre^Tam^−* and *Shank3^+/G^Cre^Tam^+* mice from this initial cross were bred to generate all experimental mice for this study: *Shank3^+/+^Cre^Tam^−*, *Shank3^+/G^Cre^Tam^−*, *Shank3^G/G^Cre^Tam^−*, *Shank3^+/+^Cre^Tam^+*, *Shank3^+/G^Cre^Tam^+*, and *Shank3^G/G^Cre^Tam^+*.

**Figure 2.**
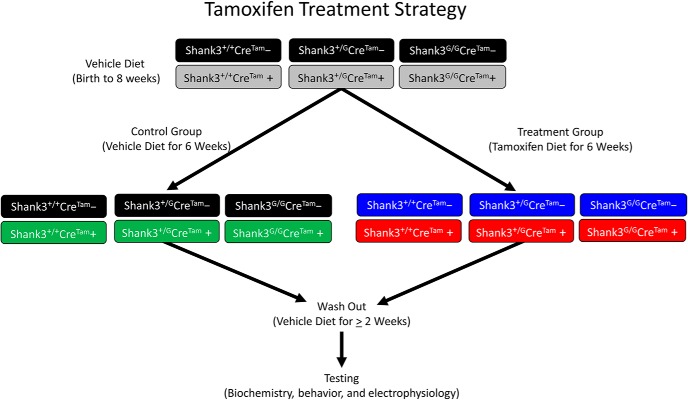
Tamoxifen treatment strategy. Experimental mice of all six genotypes (*Shank3^+/+^Cre^Tam^−*, *Shank3^+/G^Cre^Tam^−*, *Shank3^G/G^Cre^Tam^−*, *Shank3^+/+^Cre^Tam^+*, *Shank3^+/G^Cre^Tam^+*, and *Shank3^G/G^Cre^Tam^+*) were fed vehicle diet until eight weeks of age. At eight weeks, mice were separated into two treatment groups, one receiving vehicle diet for six weeks and another receiving tamoxifen diet for six weeks. Each treatment group consisted of mice from all six genotypes. After the six-week treatment, all mice were fed vehicle diet for at least a two-week wash-out period before testing. During and after testing, all mice were fed vehicle diet.

Heterozygous mice from the original *Shank3^G^* mouse line (Speed et al., 2015) were crossed with a tamoxifen-inducible *CAGGCre-ER^TM^* transgenic mouse line driven by the chicken beta actin promoter/enhancer coupled with the cytomegalovirus (CMV) immediate-early enhancer from The Jackson Laboratory (strain: B6.Cg-Tg(Cre/Esr1*)5Amc/J; Hayashi and McMahon, 2002). We refer to this tamoxifen-inducible transgene as *Cre^Tam^* throughout. This cross yielded WT (*Shank3^+/+^*) and heterozygous (*Shank3^+/G^*) mutant mice with (*Cre^Tam^+*) and without (*Cre^Tam^−*) the *Cre^Tam^* transgene. Next, heterozygous *Shank3^+/G^*mice with the *Cre^Tam^* transgene (*Shank3^+/G^Cre^Tam^+*) were crossed with heterozygous *Shank3^+/G^*mice without the *Cre^Tam^* transgene (*Shank3^+/G^Cre^Tam^−*). This final cross yielded all experimental mice (Figs. 1, 2).

Sex-matched littermates of mixed *Shank3* and *Cre^Tam^* genotypes were housed together two to four per cage on weaning at postnatal days P21–P28. Mice were kept on a 12/12 h light/dark cycle with experiments performed during the light cycle (6 A.M. to 6 P.M.). Mice were allowed free access to food and water. Mice receiving tamoxifen treatment were housed in the same room, but on a separate rack from mice receiving vehicle.

**Figure 3.**
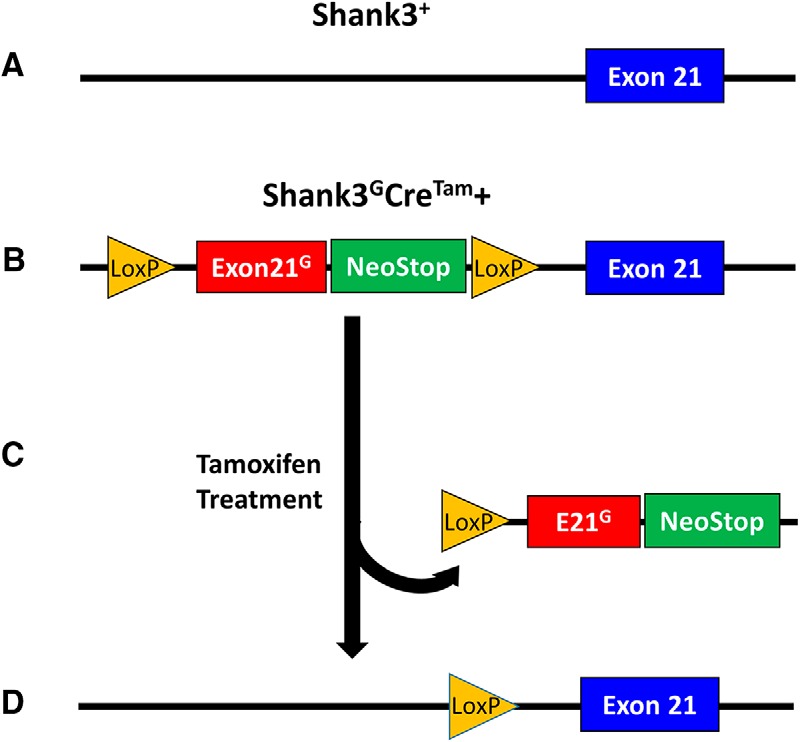
Tamoxifen-inducible *Shank3* genetic reversal strategy. The *Shank3^G^* mutation was introduced into the WT *Shank3* allele (***A***) by insertion of a neo-STOP cassette before WT exon 21 (***B***). The neo-STOP cassette was flanked by loxP sites, so that Cre-recombinase activity in the nucleus could excise the mutated S*hank3^G^* exon 21 (***C***), resulting in restoration of the WT *Shank3^+/+^* gene (***D***) and SHANK3 expression, thereby effectively reversing the *Shank3^G^* mutation.

In the *Shank3^G^Cre^Tam^+* mice, tamoxifen administration allowed Cre-recombinase to be transported into the nucleus to excise the mutated S*hank3* exon 21, resulting in WT SHANK3 expression and effectively reversing the mutation (Fig. 3). The *Shank3^G^Cre^Tam^−* mice also were subjected to behavioral, biochemical, and electrophysiological testing to identify any unanticipated effects of the *Cre^Tam^* transgene or of tamoxifen administration.

### Biochemistry

Western blottings were performed as previously described (Kouser et al., 2013). To determine rescue of SHANK3 expression following tamoxifen treatment, SHANK3 protein levels were determined by immunoblotting whole-brain tissue homogenized in artificial CSF (ACSF), 5 mM EDTA, and 1× Halt protease and phosphatase inhibitor cocktail (Thermo Scientific); 10 μg of protein was loaded per lane and blotted with an anti-SHANK3 antibody (gift of Paul Worley) and anti-β-actin antibody was used as an internal loading control. An Image Works film processor was used to develop films and the chemiluminescent signals were quantified, normalized, and analyzed using Image Studio, Microsoft Excel, and Statistica software (Version 13, Dell Inc).

### Tamoxifen administration

**Figure 4.**
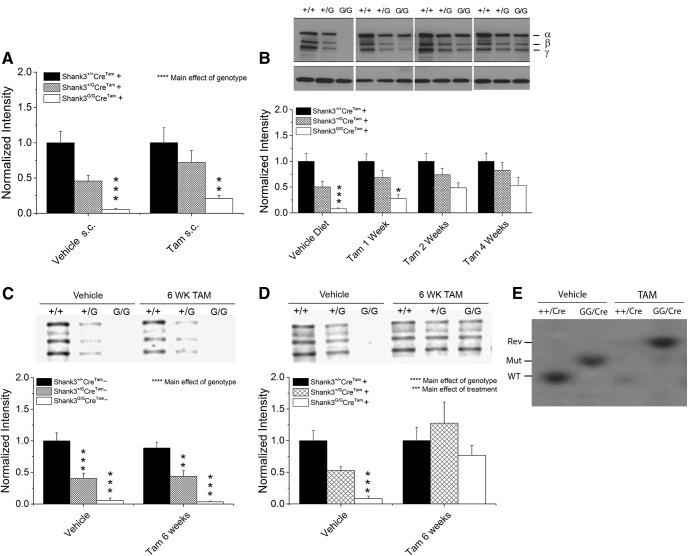
Optimization of tamoxifen treatment protocol in adult *Shank3^G^Cre^Tam^+* mice. ***A***, Quantification of Western blotting showing minimal rescue of WT SHANK3 protein expression following treatment with 4-hydroxytamoxifen (66.67 mg/kg) given once per day subcutaneously for 15 d (*n* = 7 for all treatment groups). ***B***, Representative Western blotting (top) and quantification of whole-brain lysates (bottom) showing degrees of rescue of SHANK3 protein expression after varying duration of tamoxifen diet (*n* = 7 for all treatment groups). ***C***, SHANK3 protein levels from whole-brain lysates in adult *Shank3^G^Cre^Tam^−* mice treated for six weeks with vehicle or tamoxifen diet (vehicle diet: *Shank3^+/+^ n* = 9, *Shank3^+/G^ n* = 7, *Shank3^G/G^ n* = 4; tamoxifen diet: *Shank3^+/+^ n* = 12, *Shank3^+/G^ n* = 9, *Shank3^G/G^ n* = 9). ***D***, SHANK3 protein levels from whole-brain lysates in adult *Shank3^G^Cre^Tam^+* mice treated for six weeks with vehicle diet or tamoxifen diet [vehicle diet: *Shank3^+/+^ n* = 26, *Shank3^+/G^ n* = 21, *Shank3^G/G^ n* = 15; tamoxifen diet: *Shank3^+/+^ n* = 23, *Shank3^+/G^ n* = 15, *Shank3^G/G^ n* = 18; data are normalized to the β-actin control and then to the average of WT levels with C-terminal SHANK3 antibody (JH3025)]. ***E***, Example Southern blotting of brain tissue in *Cre^Tam^*+, WT and *Shank3^G/G^* homozygous mutant mice treated with vehicle or tamoxifen (TAM; Rev = band expected following cre-mediated recombination of floxed mutant; Mut = band expected for floxed mutant before cre recombination; WT = band expected in WT mice without mutant allele); *p < 0.05, **p < 0.01, ***p < 0.001, ****p < 0.0001. Error bars represent S.E.M. in this and all subsequent figures.

The route and dose of tamoxifen administration were determined by comparing the SHANK3 protein levels in adult *Shank3^G^Cre^Tam^+* mice receiving 15-d subcutaneous injection of 4-hydroxytamoxifen or in mice being fed tamoxifen custom chow for one to four weeks (Fig. 4). Daily injections of 4-hydroxytamoxifen (66.67 mg/kg, Sigma-T176, Sigma-Aldrich) stimulated a modest increase in expression of SHANK3 in *Shank3^+/G^Cre^Tam^+* and *Shank3^G/G^Cre^Tam^+* mice compared to *Shank3^+/+^Cre^Tam^+* controls (Fig. 4*A*).

Oral treatment with tamoxifen diet (950 g/kg 16% Protein Rodent Diet, 500 mg/kg tamoxifen USP, 49.5 mg/kg sucrose, catalog #TD.130857, Envigo) provided a dose of ∼80 mg/kg/d for a 20- to 25-g mouse. Beginning at eight weeks of age, *Cre^Tam^+* and *Cre^Tam^−* mice were randomly assigned to the vehicle diet group or the tamoxifen diet group and fed *ad libitum* for one, two, or four weeks followed by a two-week washout period.

Rescue of SHANK3 expression in *Shank3^+/G^Cre^Tam^+* and *Shank3^G/G^Cre^Tam^+* mice with tamoxifen diet increased with duration of treatment from one to four weeks (Fig. 4*B*). In our final experimental design, we used a six-week treatment with tamoxifen diet to further ensure complete reversal. In the final analysis, six weeks of tamoxifen diet proved most effective in rescuing SHANK3 protein levels in *Shank3^G^Cre^Tam^+* mice (Fig. 2*C*,*D*). Thus, for the actual experiments, mice in the both groups were fed 16% Protein Rodent Diet (vehicle, Envigo) until eight weeks of age, and then the tamoxifen-treated group were fed tamoxifen chow for six weeks. Tamoxifen-treated mice were returned to their original vehicle diet for at least a two-week washout period before the start of all experiments. Mice in the control group were maintained on vehicle diet throughout the treatment and wash-out periods. Food intake and body weight were unchanged during tamoxifen administration (data not shown).

### Behavioral overview

Behavioral tests were performed during the light cycle on four cohorts: two cohorts with cre-recombinase (*Shank3^G^Cre^Tam^+*) ± tamoxifen treatment and two cohorts without cre-recombinase (*Shank3^G^Cre^Tam^****−***) ± tamoxifen treatment. All cohorts consisted of age- and sex-matched littermate progeny of heterozygous matings (Fig. 1). The appearance of unequal N’s was due to some littermate triplets (WT/het/homo) and some littermate pairs (WT/het or WT/homo) being used, with each heterozygous or homozygous mouse having at least one sex-matched littermate WT in the cohort.

All mice in each cohort were born within 12 weeks of each other. Tamoxifen dosing for the behavioral cohorts began when each pair or triplet was eight weeks of age. Tamoxifen treatment continued for six weeks before resuming a regular diet. Behavioral testing began when the mice were four to six months of age by an experimenter blind to genotype in the following order: locomotor, marble burying, rotarod, and nesting behavior. Behavioral results are not described in the order in which they were tested to simplify presentation of the data. One *Shank3^+/G^Cre^Tam^+* mouse treated with tamoxifen was found dead in its cage before marble burying behavior, so its littermate-paired, WT *Shank3^+/+^Cre^Tam^+* mouse treated with tamoxifen was excluded from the future experiments.

### Locomotor

Locomotor activity was tested by placing the mice in a fresh home cage with minimal bedding. Their activity was monitored for 2 h using photobeams linked to a computer with data acquisition software (San Diego Instruments; Powell et al., 2004) in the dark. Three-way repeated measures ANOVA (rmANOVA) was used to analyze the data with genotype and sex as between-subject factors and time as a within-subject factor.

### Marble burying

As previously described (Blundell et al., 2010), twenty marbles were evenly placed around a novel home cage with 5 cm of bedding and mice were given 30 min in the cage. After 30 min, the number of marbles buried was recorded. A marble was defined as buried when <25% of the marble was visible. The test room was well lit (∼80 lux). Data were analyzed using two-way ANOVA with genotype and sex as between-subject factors.

### Accelerating rotarod

Coordination and motor learning were tested using a rotarod as previously described (Powell et al., 2004). In a well-lit room (∼80 lux), mice were placed on a stationary rotarod (IITC Life Sciences) that was then activated and accelerated from 0 to 45 revolutions over 5 min. The latency for mice to fall off the rod or take one revolution was measured. Trials were repeated four times per day with intertrial intervals of 30 min for 2 d. Data were analyzed using three-way rmANOVA with genotype and sex as between-subject factors and trials as a within-subject factor.

### Nesting

Nesting behavior was performed in a well-lit (∼80 lux) room by first habituating the mouse to a novel, clean home-cage with ∼1.5 cm of bedding for 15 min. Then a cotton nestlet (5.5 × 5.5 × 0.5 cm) was put in the cage. Height and width of nests were measured at 30, 60, and 90 min (Etherton et al., 2009). Data were analyzed using three-way ANOVA with genotype and sex as between-subject factors and time as a within-subject factor.

### Acute slice preparation

Acute slice preparation was performed as previously described (Speed et al., 2015) with minor modifications. Sixteen-week-old male mice from both the vehicle and tamoxifen treatment groups (*Shank3^+/+^Cre^Tam^+*, *Shank3^+/+^Cre^Tam^−*, *Shank3^G/G^Cre^Tam^+*, and *Shank3^G/G^Cre^Tam^−*) were administered a lethal dose of 8% chloral hydrate (≥400 mg/kg) and perfused through the heart with ACSF. The brains were rapidly removed and cut 350–400 μm thick in ice-cold, modified dissecting ACSF on a vibrating microtome (Vibratome 3000, Leica Biosystems). Coronal slices containing hippocampus were brought to 35 ± 0.5°C for 30 min and allowed to slowly cool to room temperature, where they remained until recording at 32°C.

### Extracellular “field” electrophysiology

Field EPSPs (fEPSPs) were generated by a 100-μs biphasic pulse through a monopolar nickel dichromate stimulating electrode as previously described (Speed et al., 2015). The stimulating and glass recording electrodes (1–2 MΩ) were placed laterally in the stratum radiatum 400–500 μm apart. Data were collected using Model 2100 stimulus isolators and Model 1800 amplifiers (A-M Systems) at 10 kHz sample rate with a 1- to 5-kHz high-pass filter. Data were acquired and analyzed using the pClamp software suite (v 10.3, Molecular Devices), Prism (v 6.0, GraphPad), and Statistica (v 13, Dell Inc).

After a stable 20-min baseline was achieved at 0.05 Hz, input/output (I/O) curves were measured over a range of stimulus intensities (0–350 μA) in 50-μA increments at 0.05 Hz. The maximum slope (10–90%) of the fEPSP was analyzed at eight different stimulus intensities with five repetitions at each stimulus intensity. All recordings were performed at 32°C with an average of two to three slices per mouse. Data were analyzed using two-way rmANOVA with genotype the between-subject factor and stimulus intensity as a within-subject factor.

### Solutions

ACSF contained the following: 120 mM NaCl, 3.5 mM KCl, 1.25 mM NaH_2_PO_4_, 1 mM MgSO_4_, 26 mM NaHCO_3_, 10 mM dextrose, and 2 mM CaCl_2_. Dissection ACSF consisted of the following: 75 mM sucrose, 87 mM NaCl, 3 mM KCl, 1.25 mM NaH_2_PO_4_, 7 mM MgSO_4_, 26 mM NaHCO_3_, 20 mM dextrose, and 0.5 mM CaCl_2_. All solutions were adjusted to pH 7.4 and saturated with 95% O_2_/5% CO_2_.

### Statistics

Plotting was performed with OriginPro 2016 (OriginLab Corporation). All statistics were performed in Statistica (v 13, Dell Inc). Significance was determined at the *p* < 0.05 level. A main effect of genotype or sex was followed by a Tukey HSD test to determine significance of each group compared to control. For detailed numerical statistical results see Tables 1, 2.

**Table 1.** Detailed statistical analysis of tamoxifen treatment on WT SHANK3 protein expression

Biochemistry					
15-dTAM s.c. vsVeh s.c. (Cre+)	Two-way ANOVA	GenotypeTreatmentInteraction		*F*_(2,36)_ = 20.97*F*_(1,36)_ = 1.61*F*_(2,36)_ = 0.49	*p < 0.0001*p* = 0.2133*p* = 0.6194
(Fig. 4*A*)	Tukey HSD	Veh *Shank3^+/+^* vsVeh *Shank3^+/+^*vs Veh *Shank3^+/G^* vsVeh *Shank3^+/+^*vsVeh *Shank3^+/+^*vsVeh *Shank3^+/+^*vsTam *Shank3^+/+^*vsTam *Shank3^+/+^*vsTam *Shank3^+/G^*vs	Veh *Shank3^+/G^* Veh *Shank3^G/G^* Veh *Shank3^G/G^* Tam *Shank3^+/+^* Tam *Shank3^+/G^* Tam *Shank3^G/G^* Tam *Shank3^+/G^* Tam *Shank3^G/G^* Tam *Shank3^G/G^*		*p* = 0.0705*p = 0.0003*p* = 0.2997*p* = 1.0000*p* = 0.6835*p = 0.0024*p* = 0.6835*p = 0.0241*p* = 0.0998
One-weekTAM diet vs Veh diet (Cre+)	Two-way ANOVA	GenotypeTreatmentInteraction		*F*_(2,36)_ = 25.49*F*_(1,36)_ = 1.78*F*_(2,36)_ = 0.45	*p < 0.0001*p* = 0.1906*p* = 0.6425
(Fig. 4*B*)	Tukey HSD	Veh *Shank3^+/+^*vs Veh *Shank3^+/+^*vs Veh *Shank3^+/G^* vsVeh *Shank3^+/+^*vsVeh *Shank3^+/+^*vsVeh *Shank3^+/+^*vsTam *Shank3^+/+^*vsTam *Shank3^+/+^*vsTam *Shank3^+/G^*vs	Veh *Shank3^+/G^* Veh *Shank3^G/G^* Veh *Shank3^G/G^* Tam *Shank3^+/+^* Tam *Shank3^+/G^* Tam *Shank3^G/G^* Tam *Shank3^+/G^* Tam *Shank3^G/G^* Tam *Shank3^G/G^*		*p = 0.0447*p = 0.0002*p* = 0.1242*p* = 1.0000*p* = 0.3864*p = 0.0012*p* = 0.3864*p = 0.0012*p* = 0.1548
Two-weekTAM diet vs Veh diet (Cre+)	Two-way ANOVA	GenotypeTreatmentInteraction		*F*_(2,36)_ = 18.64*F*_(1,36)_ = 4.92*F*_(2,36)_ = 1.49	*p < 0.0001*p = 0.0329*p* = 0.2389
(Fig. 4*B*)	Tukey HSD	Veh *Shank3^+/+^*vs Veh *Shank3^+/+^*vs Veh *Shank3^+/G^* vsVeh *Shank3^+/+^*vsVeh *Shank3^+/+^*vsVeh *Shank3^+/+^*vsTam *Shank3^+/+^*vsTam *Shank3^+/+^*vsTam *Shank3^+/G^*vs	Veh *Shank3^+/G^* Veh *Shank3^G/G^* Veh *Shank3^G/G^* Tam *Shank3^+/+^* Tam *Shank3^+/G^* Tam *Shank3^G/G^* Tam *Shank3^+/G^* Tam *Shank3^G/G^* Tam *Shank3^G/G^*		*p* = 0.0523*p = 0.0002*p* = 0.1395*p* = 1.0000*p* = 0.6185*p = 0.0404*p* = 0.6185*p = 0.0404*p* = 0.6526
Four-weekTAM diet vs Veh diet (Cre+)	Two-way ANOVA	GenotypeTreatmentInteraction		*F*_(2,36)_ = 13.70*F*_(1,36)_ = 5.73*F*_(2,36)_ = 1.55	*p < 0.0001*p = 0.0220*p* = 0.2262
(Fig. 4*B*)	Tukey HSD	Veh *Shank3^+/+^*vs Veh *Shank3^+/+^*vs Veh *Shank3^+/G^* vsVeh *Shank3^+/+^*vsVeh *Shank3^+/+^*vsVeh *Shank3^+/+^*vsTam *Shank3^+/+^*vsTam *Shank3^+/+^*vsTam *Shank3^+/G^*vs	Veh *Shank3^+/G^* Veh *Shank3^G/G^* Veh *Shank3^G/G^* Tam *Shank3^+/+^* Tam *Shank3^+/G^* Tam *Shank3^G/G^* Tam *Shank3^+/G^* Tam *Shank3^G/G^* Tam *Shank3^G/G^*		*p* = 0.1104*p = 0.0004*p* = 1.0000*p* = 1.0000*p* = 0.9380*p* = 0.1530*p* = 0.9380*p* = 0.1530*p* = 0.6222
Six-weekTAM diet vsVeh diet (Cre-)	Two-way ANOVA	GenotypeTreatmentInteraction		*F*_(2,44)_ = 45.29*F*_(1,44)_ = 0.17*F*_(2,44)_ = 0.39	*p < 0.0001*p* = 0.6783*p* = 0.7010
(Fig. 4*C*)	Tukey HSD	Veh *Shank3^+/+^*vs Veh *Shank3^+/+^*vs Veh *Shank3^+/G^* vsVeh *Shank3^+/+^*vsVeh *Shank3^+/+^*vsVeh *Shank3^+/+^*vsTam *Shank3^+/+^*vsTam *Shank3^+/+^*vsTam *Shank3^+/G^*vs	Veh *Shank3^+/G^* Veh *Shank3^G/G^* Veh *Shank3^G/G^* Tam *Shank3^+/+^* Tam *Shank3^+/G^* Tam *Shank3^G/G^* Tam *Shank3^+/G^* Tam *Shank3^G/G^* Tam *Shank3^G/G^*		*p = 0.0008*p = 0.0001*p* = 0.2915*p* = 0.9236*p = 0.0007*p = 0.0001*p = 0.0046*p = 0.0001*p = 0.0255

Six-weekTAM diet vsVeh diet (Cre+)	Two-way ANOVA	GenotypeTreatmentInteraction		*F*_(2,112)_ = 7.81*F*_(1,112)_ = 14.67*F*_(2,112)_ = 4.87	*p < 0.0001*p = 0.0002*p = 0.0094
(Fig. 4*D*)	Tukey HSD	Veh *Shank3^+/+^*vs Veh *Shank3^+/+^*vs Veh *Shank3^+/G^* vsVeh *Shank3^+/+^*vsVeh *Shank3^+/+^*vsVeh *Shank3^+/+^*vsTam *Shank3^+/+^*vsTam *Shank3^+/+^*vsTam *Shank3^+/G^*vs	Veh *Shank3^+/G^* Veh *Shank3^G/G^* Veh *Shank3^G/G^* Tam *Shank3^+/+^* Tam *Shank3^+/G^* Tam *Shank3^G/G^* Tam *Shank3^+/G^* Tam *Shank3^G/G^* Tam *Shank3^G/G^*		*p* = 0.1319*p = 0.0005*p = 0.0012*p* = 1.0000*p* = 0.7763*p* = 0.8507*p* = 0.6913*p* = 0.9308*p* = 0.2261

* Significant at *P* < 0.05 level.

**Table 2.** Detailed statistical analysis of all behavior and electrophysiology performed in this study

Vehicle-treated *Shank3^G^Cre^Tam^−*				
Nest width	Three-way rmANOVA	Genotype	*F*_(2,58)_ = 4.07	^*^p = 0.0222
(Fig. 5*A*)		Time	*F*_(2,116)_ = 19.12	^*^p < 0.0001
		Sex	*F*_(1,58)_ = 0.26	*p* = 0.6119
		Genotype × time	*F*_(4,116)_ = 0.67	*p* = 0.6162
	Tukey HSD	*Shank3^+/+^Cre^Tam^−* vs	*Shank3^+/G^Cre^Tam^−*	*p* = 0.5894
		*Shank3^+/+^Cre^Tam^−* vs	*Shank3^G/G^Cre^Tam^−*	^*^p = 0.0089
		*Shank3^+/G^Cre^Tam^−* vs	*Shank3^G/G^Cre^Tam^−*	*p* = 0.1515
Nest height	Three-way rmANOVA	Genotype	*F*_(2,58)_ = 3.38	^*^p = 0.0408
(Fig. 5*B*)		Time	*F*_(2,116)_ = 33.32	^*^p < 0.0001
		Sex	*F*_(1,58)_ = 0.01	*p* = 0.9336
		Genotype × time	*F*_(4,116)_ = 0.46	*p* = 0.7621
		Genotype × sex	*F*_(2,58)_ = 1.82	*p* = 0.1710
	Tukey HSD	*Shank3^+/+^Cre^Tam^−* vs	*Shank3^+/G^Cre^Tam^−*	*p* = 0.9865
		*Shank3^+/+^Cre^Tam^−* vs	*Shank3^G/G^Cre^Tam^−*	^*^p = 0.0392
		*Shank3^+/G^Cre^Tam^− vs*	*Shank3^G/G^Cre^Tam^−*	^*^p = 0.0473
Marble burying	Two-way ANOVA	Genotype	*F*_(2,58)_ = 12.30	^*^p < 0.0001
(Fig. 5*C*)		Sex	*F*_(1,58)_ = 0.13	*p* = 0.7208
		Genotype × time	*F*_(4,116)_ = 0.67	*p* = 0.6162
	Tukey HSD	*Shank3^+/+^Cre^Tam^−* vs	*Shank3^+/G^Cre^Tam^−*	*p* = 0.4965
		*Shank3^+/+^Cre^Tam^−* vs	*Shank3^G/G^Cre^Tam^−*	^*^p = 0.0001
		*Shank3^+/G^Cre^Tam^−* vs	*Shank3^G/G^Cre^Tam^−*	^*^p = 0.0017
Locomotor	Three-way rmANOVA	Genotype	*F*_(2,58)_ = 0.47	*p* = 0.6274
(Fig. 5*D*)		Time	*F*_(23,1334)_ = 103.60	^*^p < 0.0001
		Sex	*F*_(1,58)_ = 0.82	*p* = 0.3689
		Genotype × time	*F*_(46,1334)_ = 3.37	^*^p < 0.0001
		Genotype × sex	*F*_(2,58)_ = 0.37	*p* = 0.6893
	Tukey HSD	*Shank3^+/+^Cre^Tam^−* vs	*Shank3^+/G^Cre^Tam^−*	*p* = 0.9828
		*Shank3^+/+^Cre^Tam^−* vs	*Shank3^G/G^Cre^Tam^−*	*p* = 0.5155
		*Shank3^+/G^Cre^Tam^−* vs	*Shank3^G/G^Cre^Tam^−*	*p* = 0.6796
Rotarod	Three-way rmANOVA	Genotype	*F*_(2,58)_ = 5.83	^*^p = 0.0049
(Fig. 5*E*)		Trial	*F*_(7,406)_ = 13.89	^*^p < 0.0001
		Sex	*F*_(1,58)_ = 4.31	^*^p = 0.0424
		Genotype × trial	*F*_(14,406)_ = 1.87	^*^p = 0.0282
		Genotype × sex	*F*_(2,58)_ = 0.94	*p* = 0.3970
	Tukey HSD	*Shank3^+/+^Cre^Tam^−* vs	*Shank3^+/G^Cre^Tam^−*	*p* = 0.4183
		*Shank3^+/+^Cre^Tam^−* vs	*Shank3^G/G^Cre^Tam^−*	^*^p = 0.0015
		*Shank3^+/G^Cre^Tam^−* vs	*Shank3^G/G^Cre^Tam^−*	*p* = 0.4183
fEPSP slope	Two-way rmANOVA	Genotype	*F*_(1,17)_ = 102.42	^*^p < 0.0001
(Fig. 5*F*)		Intensity	*F*_(7,119)_ = 36.51	^*^p < 0.0001
		Genotype × intensity	*F*_(7,119)_ = 2.90	^*^p = 0.0078
Fiber volley	Two-way rmANOVA	Genotype	*F*_(1,8)_ = 5.12	*p* = 0.0535
		Intensity	*F*_(7,56)_ = 69.13	^*^p < 0.0001
		Genotype × intensity	*F*_(7,56)_ = 3.51	^*^p = 0.0034
Tamoxifen-treated *Shank3^G^Cre^Tam^−*				
Nest width	Three-way rmANOVA	Genotype	*F*_(2,54)_ = 5.09	^*^p = 0.0047
(Fig. 6*A*)		Time	*F*_(2,108)_ = 23.28	^*^p < 0.0001
		Sex	*F*_(1,54)_ = 0.31	*p* = 0.5813
		Genotype × time	*F*_(4,108)_ = 1.12	*p* = 0.3527
		Genotype × sex	*F*_(2,54)_ = 0.35	*p* = 0.7070
	Tukey HSD	*Shank3^+/+^Cre^Tam^−* vs	*Shank3^+/G^Cre^Tam^−*	*p* = 0.3842
		*Shank3^+/+^Cre^Tam^−* vs	*Shank3^G/G^Cre^Tam^−*	^*^p = 0.0068
		*Shank3^+/G^Cre^Tam^−* vs	*Shank3^G/G^Cre^Tam^−*	*p* = 0.1757
Nest height	Three-way rmANOVA	Genotype	*F*_(2,54)_ = 2.47	*p* = 0.0942
(Fig. 6*B*)		Time	*F*_(2,108)_ = 39.18	^*^p < 0.0001
		Sex	*F*_(1,54)_ = 0.01	*p* = 0.9046
		Genotype × time	*F*_(4,108)_ = 1.07	*p* = 0.3753
		Genotype × sex	*F*_(2,54)_ = 0.14	*p* = 0.8719
	Tukey HSD	*Shank3^+/+^Cre^Tam^−* vs	*Shank3^+/G^Cre^Tam^−*	*p* = 0.4260
		*Shank3^+/+^Cre^Tam^−* vs	*Shank3^G/G^Cre^Tam^−*	*p* = 0.0811
		*Shank3^+/G^Cre^Tam^−* vs	*Shank3^G/G^Cre^Tam^−*	*p* = 0.6415

Marble burying	Two-way ANOVA	Genotype	*F*_(2,54)_ = 22.52	^*^p < 0.0001
(Fig. 6*C*)		Sex	*F*_(1,54)_ = 0.86	*p* = 0.3575
		Genotype × sex	*F*_(2,54)_ = 0.20	*p* = 0.8195
	Tukey HSD	*Shank3^+/+^Cre^Tam^−* vs	*Shank3^+/G^Cre^Tam^−*	*p* = 0.0776
		*Shank3^+/+^Cre^Tam^−* vs	*Shank3^G/G^Cre^Tam^−*	^*^p = 0.0001
		*Shank3^+/G^Cre^Tam^−* vs	*Shank3^G/G^Cre^Tam^−*	^*^p = 0.0003
Locomotor	Three-way rmANOVA	Genotype	*F*_(2,54)_ = 5.20	^*^p = 0.0086
(Fig. 6*D*)		Time	*F*_(23,1242)_ = 96.67	^*^p < 0.0001
		Sex	*F*_(1,54)_ = 0.56	*p* = 0.4570
		Genotype × time	*F*_(46,1242)_ = 4.55	^*^p < 0.0001
		Genotype × sex	*F*_(2,54)_ = 0.11	*p* = 0.8928
	Tukey HSD	*Shank3^+/+^Cre^Tam^−* vs	*Shank3^+/G^Cre^Tam^−*	*p* = 0.0740
		*Shank3^+/+^Cre^Tam^−* vs	*Shank3^G/G^Cre^Tam^−*	^*^p = 0.0097
		*Shank3^+/G^Cre^Tam^−* vs	*Shank3^G/G^Cre^Tam^−*	*p* = 0.6566
Rotarod	Three-way rmANOVA	Genotype	*F*_(2,54)_ = 13.62	^*^p < 0.0001
(Fig. 6*E*)		Trial	*F*_(7,378)_ = 16.98	^*^p < 0.0001
		Sex	*F*_(1,54)_ = 17.21	^*^p = 0.0001
		Genotype × trial	*F*_(14,378)_ = 2.79	^*^p = 0.0006
		Genotype × sex	*F*_(2,54)_ = 2.50	*p* = 0.0912
	Tukey HSD	*Shank3^+/+^Cre^Tam^−* vs	*Shank3^+/G^Cre^Tam^−*	^*^p = 0.0253
		*Shank3^+/+^Cre^Tam^−* vs	*Shank3^G/G^Cre^Tam^−*	^*^p = 0.0001
		*Shank3^+/G^Cre^Tam^−* vs	*Shank3^G/G^Cre^Tam^−*	^*^p = 0.0346
fEPSP slope	Two-way rmANOVA	Genotype	*F*_(1,40)_ = 8.33	^*^p = 0.0063
(Fig. 6*F*)		Intensity	*F*_(7,280)_ = 113.08	^*^p < 0.0001
		Genotype × intensity	*F*_(7,280)_ = 9.94	^*^p < 0.0001
Fiber volley	Two-way rmANOVA	Genotype	*F*_(1,19)_ = 0.49	*p* = 0.4931
		Intensity	*F*_(7,133)_ = 51.55	^*^p < 0.0001
		Genotype × intensity	*F*_(7,133)_ = 0.69	*p* = 0.6768
Tamoxifen-treated *Shank3^G^Cre^Tam^+* mice				
Nest width	Three-way rmANOVA	Genotype	*F*_(2,50)_ = 0.30	*p* = 0.7405
(Fig. 7*A*)		Time	*F*_(2,100)_ = 28.50	^*^p < 0.0001
		Sex	*F*_(1,50)_ = 0.62	*p* = 0.4336
		Genotype × time	*F*_(4,100)_ = 1.12	*p* = 0.3531
		Genotype × sex	*F*_(2,50)_ = 0.22	*p* = 0.8038
	Tukey HSD	*Shank3^+/+^Cre^Tam^+* vs	*Shank3^+/G^Cre^Tam^+*	*p* = 0.9947
		*Shank3^+/+^Cre^Tam^+* vs	*Shank3^G/G^Cre^Tam^+*	*p* = 0.7240
		*Shank3^+/G^Cre^Tam^+* vs	*Shank3^G/G^Cre^Tam^+*	*p* = 0.8221
Nest height	Three-way rmANOVA	Genotype	*F*_(2,50)_ = 0.07	*p* = 0.9335
(Fig. 7*B*)		Time	*F*_(2,100)_ = 35.62	^*^p < 0.0001
		Sex	*F*_(1,50)_ = 0.58	*p* = 0.4490
		Genotype × time	*F*_(4,100)_ = 0.48	*p* = 0.7507
		Genotype × sex	*F*_(2,50)_ = 0.17	*p* = 0.8425
	Tukey HSD	*Shank3^+/+^Cre^Tam^+* vs	*Shank3^+/G^Cre^Tam^+*	*p* = 0.9623
		*Shank3^+/+^Cre^Tam^+* vs	*Shank3^G/G^Cre^Tam^+*	*p* = 0.9767
		*Shank3^+/G^Cre^Tam^+* vs	*Shank3^G/G^Cre^Tam^+*	*p* = 0.9000
Marble burying	Two-way ANOVA	Genotype	*F*_(2,48)_ = 3.68	^*^p = 0.0326
(Fig. 7*C*)		Sex	*F*_(1,48)_ = 0.00	*p* = 0.9634
		Genotype × sex	*F*_(2,48)_ = 0.26	*p* = 0.7688
	Tukey HSD	*Shank3^+/+^Cre^Tam^+* vs	*Shank3^+/G^Cre^Tam^+*	*p* = 0.6972
		*Shank3^+/+^Cre^Tam^+* vs	*Shank3^G/G^Cre^Tam^+*	^*^p = 0.0296
		*Shank3^+/G^Cre^Tam^+* vs	*Shank3^G/G^Cre^Tam^+*	*p* = 0.6972
Locomotor	Three-way rmANOVA	Genotype	*F*_(2,50)_ = 2.12	*p* = 0.1311
(Fig. 7*D*)		Time	*F*_(23,1150)_ = 99.68	^*^p < 0.0001
		Sex	*F*_(1.50)_ = 0.49	*p* = 0.4866
		Genotype × time	*F*_(46,1150)_ = 1.82	^*^p < 0.0008
		Genotype × sex	*F*_(2,50)_ = 0.03	*p* = 0.9701
	Tukey HSD	*Shank3^+/+^Cre^Tam^+* vs	*Shank3^+/G^Cre^Tam^+*	*p* = 0.8310
		*Shank3^+/+^Cre^Tam^+* vs	*Shank3^G/G^Cre^Tam^+*	*p* = 0.1097
		*Shank3^+/G^Cre^Tam^+* vs	*Shank3^G/G^Cre^Tam^+*	*p* = 0.4023

Rotarod	Three-way rmANOVA	Genotype	*F*_(2,56)_ = 0.12	*p* = 0.8894
(Fig. 7*E*)		Trial	*F*_(7,392)_ = 17.89	^*^p < 0.0001
		Sex	*F*_(1,56)_ = 4.46	^*^p = 0.0392
		Genotype × trial	*F*_(14,392)_ = 0.76	*p* = 0.7082
		Genotype × sex	*F*_(2,56)_ = 0.29	*p* = 0.7499
	Tukey HSD	*Shank3^+/+^Cre^Tam^+* vs	*Shank3^+/G^Cre^Tam^+*	*p* = 0.9642
		*Shank3^+/+^Cre^Tam^+* vs	*Shank3^G/G^Cre^Tam^+*	*p* = 0.9906
		*Shank3^+/G^Cre^Tam^+* vs	*Shank3^G/G^Cre^Tam^+*	*p* = 0.9351
fEPSP slope	Two-way rmANOVA	Genotype	*F*_(1,26)_ = 0.34	*p* = 0.5634
(Fig. 7*F*)		Intensity	*F*_(7,182)_ = 29.71	^*^p < 0.0001
		Genotype × intensity	*F*_(7,182)_ = 6.30	^*^p < 0.0001
Fiber volley	Two-way rmANOVA	Genotype	*F*_(1,11)_ = 0.46	*p* = 0.5135
		Intensity	*F*_(7,77)_ = 24.06	^*^p < 0.0001
		Genotype × intensity	*F*_(7,77)_ = 0.16	*p* = 0.9915
Vehicle-treated *Shank3^G^Cre^Tam^+* mice				
Nest width	Three-way rmANOVA	Genotype	*F*_(2,56)_ = 2.33	*p* = 0.1065
(Fig. 8*A*)		Time	*F*_(2,112)_ = 34.73	^*^p < 0.0001
		Sex	*F*_(1,56)_ = 0.44	*p* = 0.5100
		Genotype × time	*F*_(4,112)_ = 0.91	*p* = 0.4630
		Genotype × sex	*F*_(2,56)_ = 2.13	*p* = 0.1287
	Tukey HSD	*Shank3^+/+^Cre^Tam^+* vs	*Shank3^+/G^Cre^Tam^+*	*p* = 0.6704
		*Shank3^+/+^Cre^Tam^+* vs	*Shank3^G/G^Cre^Tam^+*	*p* = 0.0637
		*Shank3^+/G^Cre^Tam^+* vs	*Shank3^G/G^Cre^Tam^+*	*p* = 0.3149
Nest height	Three-way rmANOVA	Genotype	*F*_(2,56)_ = 2.72	*p* = 0.0749
(Fig. 8*B*)		Time	*F*_(2,112)_ = 72.06	^*^p < 0.0001
		Sex	*F*_(1,56)_ = 0.59	^*^p = 0.0446
		Genotype × time	*F*_(4,112)_ = 1.32	*p* = 0.2687
		Genotype × sex	*F*_(2,56)_ = 0.82	*p* = 0.4466
	Tukey HSD	*Shank3^+/+^Cre^Tam^+* vs	*Shank3^+/G^Cre^Tam^+*	*p* = 0.6704
		*Shank3^+/+^Cre^Tam^+* vs	*Shank3^G/G^Cre^Tam^+*	*p* = 0.0637
		*Shank3^+/G^Cre^Tam^+* vs	*Shank3^G/G^Cre^Tam^+*	*p* = 0.3149
Marble burying	Two-way ANOVA	Genotype	*F*_(2,55)_ = 15.78	^*^p < 0.0001
(Fig. 8*C*)		Sex	*F*_(1,55)_ = 0.50	*p* = 0.4810
		Genotype × sex	*F*_(2,55)_ = 0.12	*p* = 0.8874
	Tukey HSD	*Shank3^+/+^Cre^Tam^+* vs	*Shank3^+/G^Cre^Tam^+*	^*^p = 0.0005
		*Shank3^+/+^Cre^Tam^+* vs	*Shank3^G/G^Cre^Tam^+*	^*^p = 0.0001
		*Shank3^+/G^Cre^Tam^+* vs	*Shank3^G/G^Cre^Tam^+*	*p* = 0.3265
Locomotor	Three-way rmANOVA	Genotype	*F*_(2,56)_ = 2.48	*p* = 0.0926
(Fig. 8*D*)		Time	*F*_(23,1288)_ = 101.49	^*^p < 0.0001
		Sex	*F*_(1,56)_ = 1.98	*p* = 0.1644
		Genotype × time	*F*_(46,1288)_ = 2.57	^*^p < 0.0001
		Genotype × sex	*F*_(2,56)_ = 0.11	*p* = 0.8946
	Tukey HSD	*Shank3^+/+^Cre^Tam^+* vs	*Shank3^+/G^Cre^Tam^+*	*p* = 0.0842
		*Shank3^+/+^Cre^Tam^+* vs	*Shank3^G/G^Cre^Tam^+*	*p* = 0.9893
		*Shank3^+/G^Cre^Tam^+* vs	*Shank3^G/G^Cre^Tam^+*	*p* = 0.1944
Rotarod	Three-way rmANOVA	Genotype	*F*_(1,56)_ = 0.12	*p* = 0.8894
(Fig. 8*E*)		Trial	*F*_(7,392)_ = 17.89	^*^p < 0.0001
		Sex	*F*_(1,56)_ = 4.46	^*^p = 0.0392
		Genotype × trial	*F*_(14,392)_ = 0.76	*p* = 0.7082
		Genotype × sex	*F*_(2,56)_ = 0.29	*p* = 0.7499
	Tukey HSD	*Shank3^+/+^Cre^Tam^+* vs	*Shank3^+/G^Cre^Tam^+*	*p* = 0.9642
		*Shank3^+/+^Cre^Tam^+* vs	*Shank3^G/G^Cre^Tam^+*	*p* = 0.9906
		*Shank3^+/G^Cre^Tam^+* vs	*Shank3^G/G^Cre^Tam^+*	*p* = 0.9351
fEPSP slope	Two-way rmANOVA	Genotype	*F*_(1,30)_ = 0.26	*p* = 0.6130
(Fig. 8*F*)		Intensity	*F*_(7,210)_ = 52.49	^*^p < 0.0001
		Genotype × intensity	*F*_(7,210)_ = 0.45	*p* = 0.8699
Fiber volley	Two-way rmANOVA	Genotype	*F*_(1,15)_ = 1.80	*p* = 0.1995
		Intensity	*F*_(7,105)_ = 155.78	^*^p < 0.0001
		Genotype × intensity	*F*_(7,105)_ = 2.96	^*^p = 0.0072

Effect of CAG-Cre^Tam^ on *Shank3^+/+^* mice				
Nest height	Three-way rmANOVA	CAG-Cre^Tam^	*F*_(1,49)_ = 3.12	*p* = 0.0837
		Time	*F*_(2,98)_ = 44.08	^*^p < 0.0001
		Sex	*F*_(1,49)_ = 3.83	*p* = 0.0559
		CAG-Cre^Tam^ × time	*F*_(2,98)_ = 1.65	*p* = 0.1972
		CAG-Cre^Tam^ × sex	*F*_(2,549_ = 0.19	*p* = 0.6674
Nest width	Three-way rmANOVA	CAG-Cre^Tam^	*F*_(1,49)_ = 0.08	*p* = 0.7752
		Time	*F*_(2,98)_ = 27.22	^*^p < 0.0001
		Sex	*F*_(1,49)_ = 3.37	*p* = 0.0725
		CAG-Cre^Tam^ × time	*F*_(2,98)_ = 1.28	*p* = 0.2839
		CAG-Cre^Tam^ × sex	*F*_(2,49)_ = 0.299	*p* = 0.5872
Marble	Two-way ANOVA	CAG-Cre^Tam^	*F*_(1,49)_ = 0.34	^*^p = 0.5654
Burying		Sex	*F*_(1,49)_ = 2.93	*p* = 0.0934
		CAG-Cre^Tam^ × sex	*F*_(1,49)_ = 0.43	*p* = 0.5127
Locomotor	Three-way rmANOVA	Genotype	*F*_(1,49)_ = 16.00	^*^p = 0.0002
		Time	*F*_(23,1127)_ = 110.93	^*^p < 0.0001
		Sex	*F*_(1,49)_ = 2.38	*p* = 0.1293
		CAG-Cre^Tam^ × time	*F*_(23,1127)_ = 1.46	*p* = 0.0749
		CAG-Cre^Tam^ × sex	*F*_(1,49)_ = 0.19	*p* = 0.6626
Rotarod	Three-way rmANOVA	CAG-Cre^Tam^	*F*_(1,49)_ = 0.21	*p* = 0.6472
		Trial	*F*_(7,343)_ = 19.90	^*^p < 0.0001
		Sex	*F*_(1,49)_ = 1.99	*p* = 0.1651
		CAG-Cre^Tam^ × trial	*F*_(7,343)_ = 0.76	*p* = 0.6180
		CAG-Cre^Tam^ × sex	*F*_(1,49)_ = 0.79	*p* = 0.5973
fEPSP slope	Two-way rmANOVA	CAG-Cre^Tam^	*F*_(1,24)_ = 86.39	^*^p < 0.0001
(Fig. 9*A*)		Intensity	*F*_(7,168)_ = 58.00	^*^p < 0.0001
		CAG-Cre^Tam^ × intensity	*F*_(7,168)_ = 3.60	^*^p = 0.0012
Fiber volley	Two-way rmANOVA	CAG-Cre^Tam^	*F*_(1,9)_ = 0.69	*p* = 0.4270
		Intensity	*F*_(7,63)_ = 56.18	^*^p < 0.0001
		CAG-Cre^Tam^ × intensity	*F*_(7,63)_ = 2.95	^*^p = 0.0098
Effect of CAG-Cre^Tam^ on *Shank3^G/^* ^G^ mice				
Nest height	Three-way rmANOVA	CAG-Cre^Tam^	*F*_(1,30)_ = 0.33	*p* = 0.5693
		Time	*F*_(2,60)_ = 21.20	^*^p < 0.0001
		Sex	*F*_(1,30)_ = 1.94	*p* = 0.1736
		CAG-Cre^Tam^ × time	*F*_(2,60)_ = 0.38	*p* = 0.6865
		CAG-Cre^Tam^ × sex	*F*_(2,60)_ = 0.44	*p* = 0.5132
Nest width	Three-way rmANOVA	CAG-Cre^Tam^	*F*_(1,30)_ = 0.12	*p* = 0.7331
		Time	*F*_(2,60)_ = 20.06	^*^p < 0.0001
		Sex	*F*_(1,30)_ = 3.37	*p* = 0.0762
		CAG-Cre^Tam^ × time	*F*_(2,60)_ = 0.08	*p* = 0.9219
		CAG-Cre^Tam^ × sex	*F*_(2,60)_ = 0.02	*p* = 0.8884
Marble	Two-way ANOVA	CAG-Cre^Tam^	*F*_(1,30)_ = 0.00	*p =* 0.9828
Burying		Sex	*F*_(1,30)_ = 0.40	*p* = 0.5294
		CAG-Cre^Tam^ × sex	*F*_(1,30)_ = 1.03	*p* = 0.3179
Locomotor	Three-way rmANOVA	CAG-Cre^Tam^	*F*_(1,30)_ = 11.83	^*^p = 0.0017
		Time	*F*_(23,690)_ = 36.91	^*^p < 0.0001
		Sex	*F*_(1,30)_ = 0.05	*p* = 0.8193
		CAG-Cre^Tam^ × time	*F*_(23,690)_ = 0.89	*p* = 0.6145
		CAG-Cre^Tam^ × sex	*F*_(1,30)_ = 0.18	*p* = 0.6777
Rotarod	Three-way rmANOVA	CAG-Cre^Tam^	*F*_(1,30)_ = 5.99	^*^p = 0.0205
		Trial	*F*_(7,210)_ = 4.39	^*^p < 0.0001
		Sex	*F*_(1,30)_ = 1.99	*p* = 0.1682
		CAG-Cre^Tam^ × trial	*F*_(7,210)_ = 1.00	*p* = 0.4323
		CAG-Cre^Tam^ × sex	*F*_(1,30)_ = 1.42	*p* = 0.2422
fEPSP slope	Two-way rmANOVA	CAG-Cre^Tam^	*F*_(1,23)_ = 0.58	*p* = 0.4527
(Fig. 9*B*)		Intensity	*F*_(7,161)_ = 33.71	^*^p < 0.0001
		CAG-Cre^Tam^ × intensity	*F*_(7,161)_ = 0.173	*p* = 0.9904
Fiber volley		CAG-Cre^Tam^	*F*_(1,8)_ = 0.20	*p* = 0.6695
		Intensity	*F*_(7,56)_ = 28.25	^*^p < 0.0001
		CAG-Cre^Tam^ × intensity	*F*_(7,56)_ = 2.67	^*^p = 0.0184

* Significant at 0.05 level.

## Results

### Treatment of *Shank3^G^Cre^Tam^+* mice with tamoxifen diet results in rescued expression of SHANK3 protein in whole-brain lysates

In tamoxifen-treated, cre-positive, heterozygous and homozygous mice, the level of SHANK3 protein was rescued effectively to WT levels (Fig. 4*C*,*D*). Statistics for Figure 4 are summarized in Table 1. Adult (eight-week-old) mice were assigned to either the tamoxifen treatment group, that received tamoxifen diet for six weeks, or the control group, that continued to receive vehicle diet. After the six-week treatment period, both groups received vehicle diet for at least a two-week wash-out period before testing. This tamoxifen treatment protocol resulted in nearly complete biochemical rescue of SHANK3 expression with no significant difference in SHANK3 protein expression levels among tamoxifen-treated, mutant *Shank3^G/G^Cre^Tam^+* mice (adult-induced genetic reversal) compared to each of the several WT groups (vehicle-treated *Shank3^+/+^Cre^Tam^+*, tamoxifen-treated *Shank3^+/+^Cre^Tam^+*, vehicle-treated *Shank3^+/+^Cre^Tam^−*, and tamoxifen-treated *Shank3^+/+^Cre^Tam^−*). Similarly, no significant difference was observed among tamoxifen-treated heterozygous *Shank3^+/G^Cre^Tam^+* mice and all other WT groups. Consistent with our expectations and previous findings (Speed et al., 2015), all other heterozygous (vehicle-treated *Shank3^+/G^Cre^Tam^+*, vehicle-treated *Shank3^+/G^Cre^Tam^−*, and tamoxifen-treated *Shank3^+/G^Cre^Tam^−*) and homozygous (vehicle-treated *Shank3^G/G^Cre^Tam^+*, vehicle-treated *Shank3^G/G^Cre^Tam^−*, and tamoxifen-treated *Shank3^G/G^ Cre^Tam^−*) groups without genetic reversal demonstrated an ∼50% reduction (heterozygotes) or nearly complete loss (homozygotes) of SHANK3 protein expression. Importantly, tamoxifen diet had no effect on SHANK3 protein expression levels in cre-negative mice (*Shank3^+/G^Cre^Tam^−* and *Shank3^G/G^Cre^Tam^−*; Fig. 4*C*).

### Replication of behavioral and synaptic phenotypes in vehicle-treated *Shank3^G^Cre^Tam^−* mice

**Figure 5.**
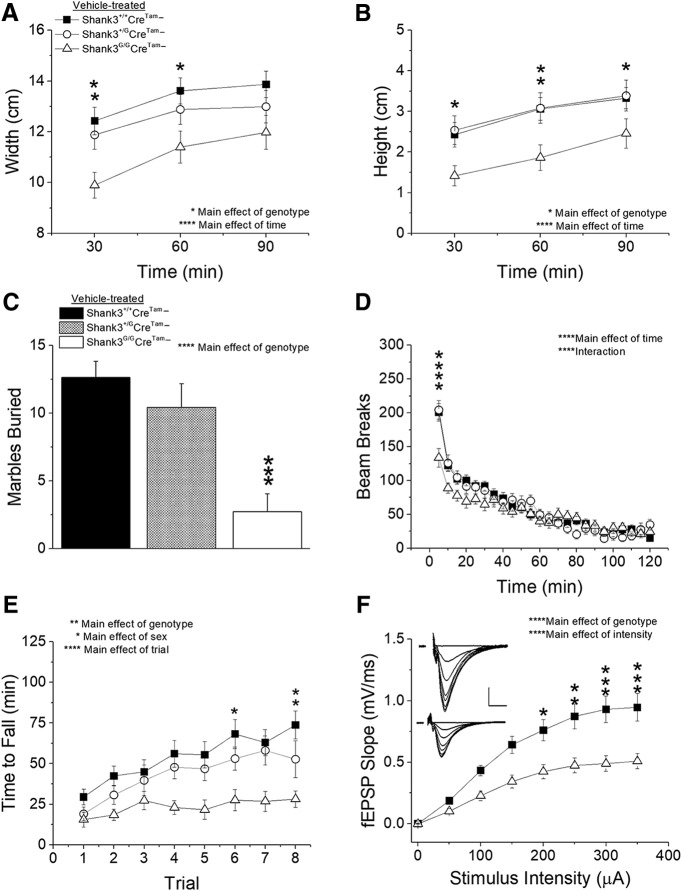
Vehicle-treated *Shank3^G/G^Cre^Tam^−* mice exhibit clear behavioral and physiologic phenotypes. *Shank3^G/G^Cre^Tam^−* mice exhibit a novelty avoidance phenotype by building smaller nests in both nest width (***A***) and height (***B***) over a 90-min period, burying fewer marbles over a 30-min period (***C***), and exhibiting hypoactivity within the first 5 min of the open field test (***D***) compared to *Shank3^+/+^Cre^Tam^−* controls. ***E***, Rotarod testing demonstrates that motor learning and coordination are decreased in *Shank3^G/G^Cre^Tam^−* mice compared to controls. *Shank3^+/+^ n* = 27, *Shank3^+/G^ n* = 18, *Shank3^G/G^ n* = 19. ***F***, Synaptic transmission in the hippocampus is impaired in *Shank3^G/G^Cre^Tam^−* mice with decreased fEPSP in response to stimulus intensity compared to *Shank3^+/+^Cre^Tam^−* mice. Inset, Average of five consecutive raw traces at stimulus intensities 0–350 μA in 50-μA steps from *Shank3^+/+^*(top) and *Shank3^G/G^* (bottom) mice; scale bar = 0.5 mV, 5 ms. *Shank3^+/+^ n* = 10 slices from five mice, *Shank3^G/G^ n* = 9 slices from three mice; *p < 0.05, **p < 0.01, ***p < 0.001, ****p < 0.0001.

*Shank3^G^* mice (Speed et al., 2015), as well as Shank3^ΔC^ mice (Kouser et al., 2013), show what we refer to as an altered response to novelty phenotype that we have now replicated in our vehicle-treated, cre-negative, *Shank3^G/G^Cre^Tam^−* mice. We tested *Shank3^+/+^Cre^Tam^−*, *Shank3^+/G^Cre^Tam^−,* and *Shank3^G/G^Cre^Tam^−* mice in a nest-building task. Homozygous *Shank3^G/G^Cre^Tam^−* mutant mice showed decreased nest width (Fig. 5*A*) and height (Fig. 5*B*) over a 90-min period compared to WT *Shank3^+/+^Cre^Tam^−* controls. We also replicated our previously demonstrated phenotype (Speed et al., 2015) in a marble burying task with *Shank3^G/G^Cre^Tam^−* mice showing a significant decrease in the number of marbles buried compared to WT controls (Fig. 5*C*). Complete statistical analysis for these and all subsequent experiments is detailed in Table 2.

In accordance with the altered response to novelty phenotype, *Shank3^G/G^Cre^Tam^−* mice also demonstrated significantly decreased initial locomotor activity in a novel environment (Fig. 5*D*). As we previously demonstrated with both *Shank3^G/G^* (Speed et al., 2015) and *Shank3^ΔC^* (Kouser et al., 2013) mice, there is no main effect of genotype on the total number of beam breaks. Tukey *post hoc* analysis, however, revealed that *Shank3^G/G^Cre^Tam^−* mice are hypoactive during the first 5 min in the novel environment compared to *Shank3^+/G^Cre^Tam^−* and *Shank3^+/+^Cre^Tam^−* littermates (Fig. 5*D*).

We next examined motor coordination and learning on the accelerating rotarod in *Shank3^G^Cre^Tam^−* mice. A decreased motor coordination phenotype was previously observed in these and related *Shank3* mutant mice (Speed et al., 2015; Kouser et al., 2013). *Shank3^G/G^Cre^Tam^−* mice exhibited a significant decrease in coordination on the rotarod, as well as a decrease in motor learning, indicated by an overall main effect of genotype and an interaction between genotype and trial (Fig. 5*E*). We also identified a main effect of sex that revealed longer latencies to fall in female mice than in males. No interaction between genotype and sex was observed, but overall, females performed better than males.

Finally, we investigated basal synaptic transmission in the hippocampus using extracellular electrophysiology in acute brain slices from male, vehicle-treated WT *Shank3^+/+^Cre^Tam^−* and homozygous *Shank3^G/G^Cre^Tam^−* mice. We found a significant decrease in *Shank3^G/G^Cre^Tam^−* synaptic transmission compared to that of *Shank3^+/+^Cre^Tam^−* mice, with a significant decrease in the I/O relationship of stimulus intensity to the slope of the fEPSP (Fig. 5*F*) at CA3 Schaffer-collateral to area CA1 synapses. Main effects of both genotype and stimulus intensity are observed along with a significant interaction between genotype and stimulus intensity. Overall, *Shank3^G/G^Cre^Tam^−* mice exhibited decreased basal synaptic strength compared to controls at stimulus intensities >50 μA and reached a 44% weaker maximum fEPSP slope. Fiber volley amplitude was not affected. Thus, breeding with the *Cre^Tam^* transgenic mouse line did not alter the previously observed phenotypes in *Shank3^G/G^* mice (Speed et al., 2015).

### Replication of behavioral and synaptic phenotypes in tamoxifen-treated, *Shank3^G/G^Cre^Tam^*− mice

**Figure 6.**
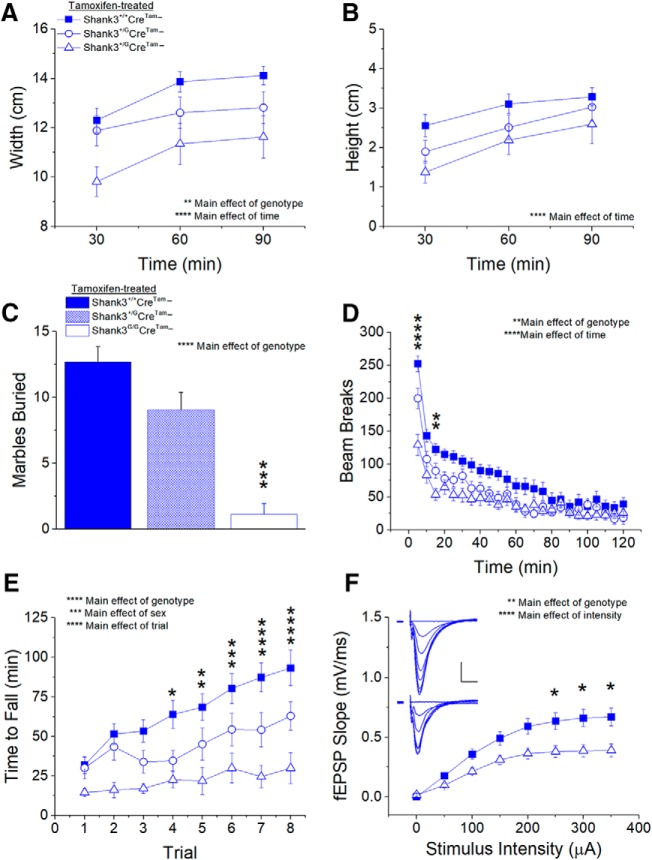
Behavioral and synaptic phenotypes in *Shank3^G/G^Cre^Tam^−* mice are not rescued by treatment with six weeks of tamoxifen diet. Nesting behavior is not rescued by tamoxifen treatment in *Shank3^G/G^Cre^Tam^−* mice with regard to nest width (***A***), although there is no main effect of genotype with regard to nest height (***B***). Marble burying (***C***) remains impaired in tamoxifen-treated *Shank3^G/G^Cre^Tam^−* mice with decreased number of marbles buried compared to controls. Locomotor activity also remains decreased in tamoxifen-treated *Shank3^G/G^Cre^Tam^−* mice at the start of the open field test (***D***). Latency to fall off the rotarod (***E***) remains decreased in tamoxifen-treated *Shank3^G/G^Cre^Tam^−* mice compared to controls. *Shank3^+/+^ n* = 25, *Shank3^+/G^ n* = 19, *Shank3^G/G^ n* = 16. ***F***, Tamoxifen treatment does not rescue synaptic transmission in *Shank3^G/G^Cre^Tam^−* mice. Inset, Average of five consecutive traces at each stimulus intensity 0–350 μA in 50-μA steps from *Shank3^+/+^* (top) and *Shank3^G/G^* (bottom) mice treated with six weeks of tamoxifen diet; scale bar = 0.25 mV, 5 ms. *Shank3^+/+^ n* = 22 slices from eight mice, *Shank3^G/G^ n* = 20 slices from six mice; *p < 0.05, **p < 0.01, ***p < 0.001, ****p < 0.0001.

As expected, tamoxifen treatment did not rescue behavioral phenotypes in Cre-negative, mutant *Shank3^G/G^Cre^Tam^−* mice compared to tamoxifen-treated, WT, Cre-negative *Shank3^+/+^Cre^Tam^−* control mice. Nest width was significantly decreased in tamoxifen-treated *Shank3^G/G^Cre^Tam^−* mice compared to controls, with a main effect of genotype present in nest width (Fig. 6*A*). The decrease in nest height of *Shank3^G/G^Cre^Tam^−* mice, however, did not reach statistical significance (Fig. 6*B*). Similarly, in the marble burying task, tamoxifen-treated *Shank3^G/G^Cre^Tam^−* mice buried fewer marbles over a 30-min period than did tamoxifen-treated *Shank3^+/+^Cre^Tam^−* controls (Fig. 6*C*).

The same lack of effect of tamoxifen on behavioral phenotypes in *Shank3^G/G^Cre^Tam^−* mice was observed in locomotor, motor learning, and motor coordination tasks. In locomotor activity (Fig. 6*D*), there was a main effect of genotype due to a decrease in the number of beam breaks by tamoxifen-treated *Shank3^G/G^Cre^Tam^−* mice compared to *Shank3^+/G^Cre^Tam^−* and *Shank3^+/+^Cre^Tam^−* mice over the 120-min testing period. As with vehicle-treated mice without the *Cre^Tam^* transgene (Fig. 5*D*), Tukey *post hoc* analysis showed that this effect was primarily due to hypoactivity of *Shank3^G/G^Cre^Tam^−* mice in the first 5 min of testing. We also observed a main effect of genotype with both *Shank3^+/G^Cre^Tam^−* and *Shank3^G/G^Cre^Tam^−* mice demonstrating decreased coordination compared to WT *Shank3^+/+^Cre^Tam^−* controls (Fig. 6*E*).

Tamoxifen treatment also had no effect on the reduction in synaptic transmission in cre-negative *Shank3^G/G^Cre^Tam^−* mice compared to WT. Synaptic strength in response to increasing stimulus intensity (Fig. 6*F*) remained significantly decreased in tamoxifen-treated *Shank3^G/G^Cre^Tam^−* mice compared to WT *Shank3^+/+^Cre^Tam^−* controls, particularly at higher stimulus intensities. Fiber volley amplitude was similar between *Shank3^+/+^Cre^Tam^−* and *Shank3^G/G^Cre^Tam^−* mice, suggesting no effect on presynaptic excitability or axon number. As predicted, these behavioral and synaptic data demonstrated that tamoxifen treatment in the absence of the *Cre^Tam^* transgene did not rescue deficits in irreversible *Shank3^G/G^Cre^Tam^−* mice.

### Apparent partial genetic rescue of behavioral and physiologic phenotypes in tamoxifen-treated, reversible *Shank3^G/G^Cre^Tam^+* mice

**Figure 7.**
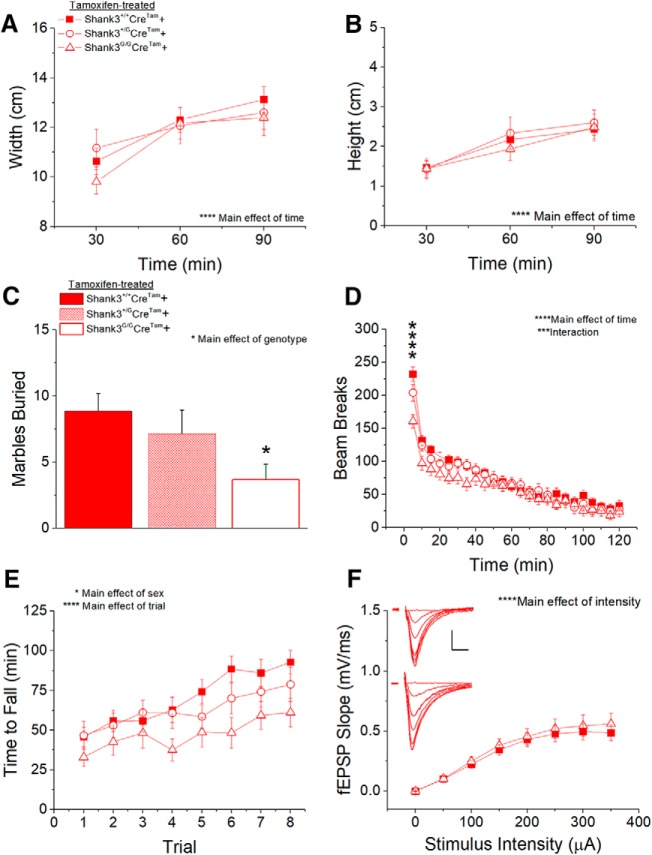
Incomplete genetic rescue of behavioral and physiologic phenotypes in tamoxifen-treated *Shank3^G/G^Cre^Tam^+* mice. There is no main effect of genotype on nest width (***A***) or height (***B***) in tamoxifen-treated *Shank3^G/G^Cre^Tam^+* mice, suggesting successful rescue of the *Shank3^G/G^* nest-building phenotype. ***C***, The *Shank3^G/G^* marble-burying phenotype is not rescued in tamoxifen-treated *Shank3^G/G^Cre^Tam^+* mice, nor is the initial hypoactivity observed in the open field test (***D***). Tamoxifen treatment of *Shank3^G/G^Cre^Tam^+* mice does successfully eliminate the main effects of genotype in the time to fall from the rotarod. *Shank3^+/+^ n* = 23, *Shank3^+/G^ n* = 15, *Shank3^G/G^ n* = 18 (***E***). ***F***, There is no main effect of genotype on fEPSP slope over a range of stimulus intensities in tamoxifen-treated *Shank3^+/+^Cre^Tam^+* and *Shank3^G/G^Cre^Tam^+* mice. Inset, Average of five consecutive traces at each stimulus intensity 0–350 μA in 50-μA steps from *Shank3^+/+^* (top) and *Shank3^G/G^* (bottom) mice treated with six weeks of tamoxifen diet; scale bar = 0.25 mV, 5 ms. *Shank3^+/+^ n* = 16 slices from eight mice, *Shank3^G/G^ n* = 12 slices from six mice; *p < 0.05, ***p < 0.0001, ****p < 0.00001.

*Shank3^G^Cre^Tam^+* mice treated with six weeks of tamoxifen diet exhibited partial rescue of novelty avoidance phenotypes identified in *Shank3^G^Cre^Tam^−* mice. In the nest-building test, there was no longer a main effect of genotype on nest width (Fig. 7*A*) or height (Fig. 7*B*) over a 30-min period. However, in the marble-burying task (Fig. 7*C*), tamoxifen treatment did not rescue the main effect of genotype on number of marbles buried. *Shank3^G/G^Cre^Tam^+* mice buried 57% fewer marbles than WT *Shank3^+/+^Cre^Tam^+* mice also treated with tamoxifen diet. Tamoxifen treatment also failed to rescue the hypoactive locomotor response to a novel environment seen in vehicle-treated (Fig. 5*D*) and tamoxifen-treated (Fig. 6*D*) *Shank3^G^Cre^Tam^−* mice. In the first 5 min of the open field test (Fig. 7*D*), Tukey *post hoc* analysis identified a significant 31% decrease in locomotor activity in *Shank3^G/G^Cre^Tam^+* mice compared to WT *Shank3^+/+^Cre^Tam^+* controls.

Testing on the rotarod (Fig. 7*E*) revealed that motor learning or coordination was also rescued; no significant effect of genotype was apparent with tamoxifen treatment of *Shank3^G/G^Cre^Tam^+* mice. That said, a trend toward decreased motor coordination/learning was still evident in this experiment. After tamoxifen treatment, there was no main effect of genotype, but significant effects of trial and sex were apparent, with females again outperforming males and with no interaction between genotype and sex.

Synaptic transmission was apparently rescued in *Shank3^G/G^Cre^Tam^+* mice treated with tamoxifen diet for six weeks. fEPSP slope in response to increasing stimulus intensity (Fig. 7*F*) and maximum fEPSP slope (*Shank3^+/+^*: 0.49 ± 0.07 mV, *Shank3^G/G^*: 0.56 ± 0.09 mV) were comparable between tamoxifen-treated *Shank3^G/G^Cre^Tam^+* mice and tamoxifen-treated WT *Shank3^+/+^Cre^Tam^+* controls, as were fiber volley amplitudes.

### Vehicle-treated *Shank3^G/G^Cre^Tam^+* controls demonstrate that apparent partial genetic reversal with tamoxifen is uninterpretable

We performed critical controls throughout this study to account for potential off-target effects of the *Shank3^G^* gene and of tamoxifen treatment on autism-associated behaviors and synaptic transmission. Control experiments resulted in robust replication of the original behavioral and synaptic deficits following both vehicle (Fig. 5) and tamoxifen (Fig. 6) treatment on the same genetic background in cre-negative *Shank3^G^Cre^Tam^−* mice.

Thus, one is tempted to conclude that genetic reversal of SHANK3 expression in adult mice leads to reversal of both synaptic dysfunction and at least one behavioral difference in *Shank3^G/G^* mice (Fig. 7). Such a conclusion would have potential ramifications for development of future treatments and for timing of such interventions in future clinical trials. As a final, critical control, we also examined vehicle-treated, *Shank3^G/G^Cre^Tam^*+ mice with the expectation that they too would replicate previously published (Kouser et al., 2013; Speed et al., 2015) and currently replicated (Figs. 5, 6), behavioral and synaptic phenotypes. This was not the case.

**Figure 8.**
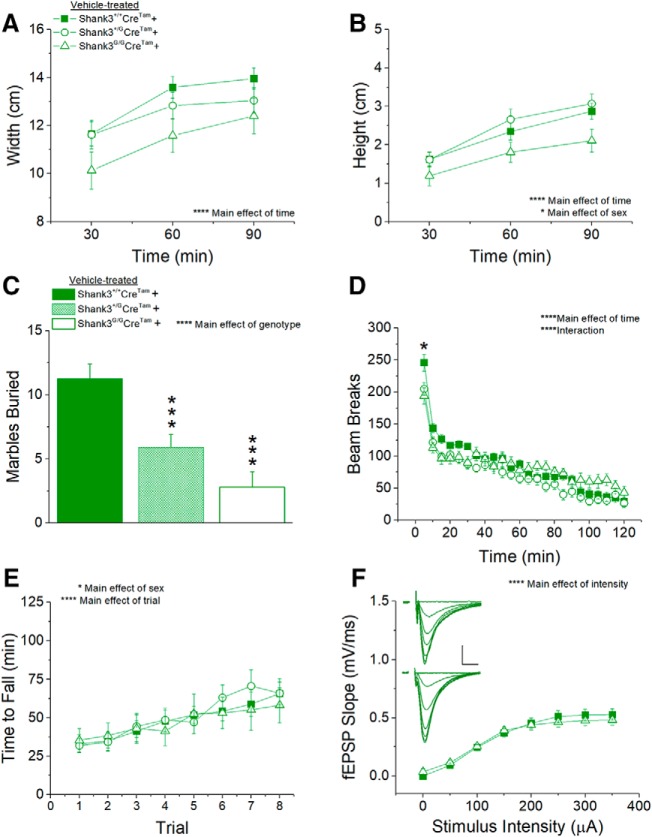
Unexpected, partial genetic “rescue” of behavioral and synaptic phenotypes in vehicle-treated *Shank3^G/G^Cre^Tam^+* mice. The decreases in nest width (***A***) and height (***B***) in *Shank3^G/G^* mice are rescued in vehicle-treated *Shank3^G/G^Cre^Tam^+* mice. The *Shank3^G/G^*marble-burying phenotype (***C***) and initial locomotor hypoactivity in the open field test (***D***) are not rescued in vehicle-treated *Shank3^G/G^Cre^Tam^+* mice. ***E***, There is no main effect of genotype in time to fall from the rotarod in vehicle-treated *Shank3^G^Cre^Tam^+* mice. *Shank3^+/+^ n* = 26, *Shank3^+/G^ n* = 21, *Shank3^G/G^ n* = 15. ***F***, There is no main effect of genotype on fEPSP slope in response to a range of stimulus intensities. Inset, Average of five consecutive traces at each stimulus intensity 0–350 μA in 50-μA steps from *Shank3^+/+^* (top) and *Shank3^G/G^* (bottom) mice treated with vehicle diet; scale bar = 0.25 mV, 5 ms. *Shank3^+/+^ n* = 16 slices from six mice, *Shank3^G/G^ n* = 16 slices from five mice; *p < 0.05, ***p < 0.0001, ****p < 0.00001.

Surprisingly, vehicle-treated, mutant *Shank3^G/G^Cre^Tam^+* mice showed no difference in nest width (Fig. 8*A*) or height (Fig. 8*B*) compared to vehicle-treated, WT *Shank3^+/+^Cre^Tam^*+ controls (a behavior that appeared to have been rescued by tamoxifen treatment in Fig. 7*A*,*B*). Marble burying (Fig. 8*C*) and initial locomotor activity (Fig. 8*D*) were significantly impaired in vehicle-treated *Shank3^G/G^Cre^Tam^+* mice, as expected, replicating the robust *Shank3^G^*phenotypes identified in the original *Shank3^G^* and *Shank3^G^Cre^Tam^−*lines (and the lack of rescue with tamoxifen treatment in Fig. 7*C*,*D*). However, motor coordination and learning on the rotarod (Fig. 8*E*) were not significantly different between vehicle-treated *Shank3^G/G^Cre^Tam^*+ and *Shank3^+/+^Cre^Tam^+* mice. Perhaps more surprisingly, synaptic transmission (Fig. 8*F*) was not significantly different between vehicle-treated, mutant *Shank3^G/G^Cre^Tam^*+ and WT *Shank3^+/+^Cre^Tam^+* mice. The lack of significant difference in the vehicle-treated *Shank3^G/G^Cre^Tam^+* mice from controls in nest-building, motor learning/coordination, and synaptic transmission makes it difficult to conclude that the apparent reversal in tamoxifen-treated *Shank3^G/G^Cre^Tam^*+ mice in nest building and synaptic transmission is due to rescue of SHANK3 protein expression.

**Figure 9.**
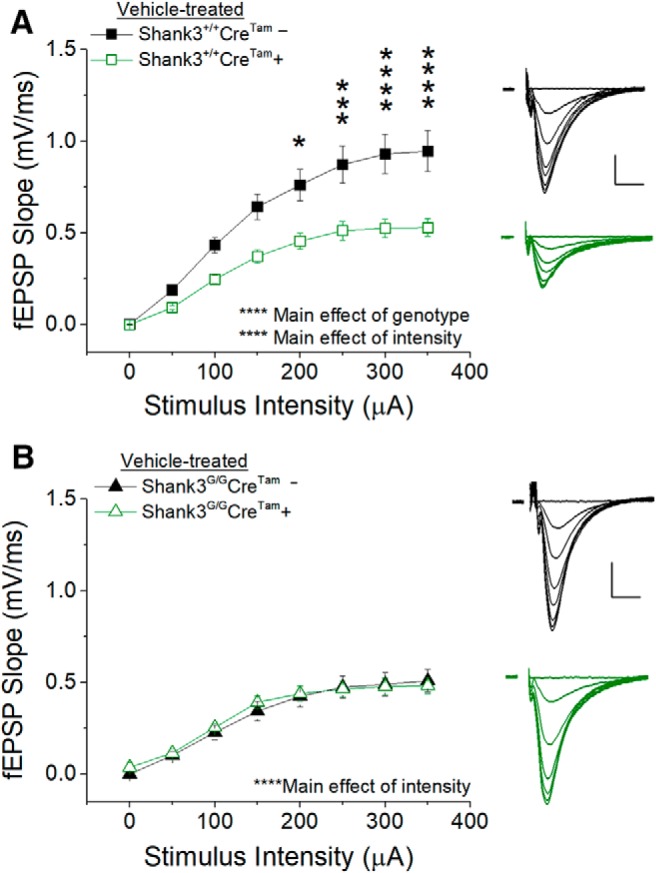
Effect of the *Cre^Tam^* transgene on synaptic physiology in WT *Shank3^+/+^* and mutant *Shank3^G/G^* mice. The *Cre^Tam^* transgene causes a dramatic decrease in the relationship between stimulus intensity and fEPSP slope (***A***) in WT *Shank3^+/+^* mice. Inset, Average of five consecutive raw traces at each stimulus intensity from 0 to 350 μA in 50-μA steps from *Shank3^+/+^Cre^Tam^−* mice (top) and *Shank3^+/+^Cre^Tam^+* mice (bottom); scale bar = 0.25 mV, 5 ms. *Shank3^+/+^Cre^Tam^− n* = 10 slices from four mice, *Shank3^+/+^Cre^Tam^+ n* = 16 slices from six mice. ***B***, The relationship between stimulus intensity and fEPSP slope in mutant *Shank3^G/G^* mice is unchanged with *Cre^Tam^* transgene expression. Inset, Average of five consecutive raw traces at each stimulus intensity from 0 to 350 μA in 50-μA steps from *Shank3^G/G^Cre^Tam^−* mice (top) and *Shank3^G/G^Cre^Tam^+* mice (bottom); scale bar =0.25 mV, 5 ms. *Shank3^G/G^Cre^Tam^− n* = 9 slices from three mice, *Shank3^G/G^Cre^Tam^+ n* = 16 slices from five mice; *p < 0.05, **p < 0.01, ***p < 0.001, ****p < 0.0001.

One possible remaining explanation for the lack of a synaptic phenotype in our Cre-positive mice treated with either vehicle (Fig. 8) or tamoxifen (Fig. 7) could be that the Cre transgene affects synaptic transmission in WT mice. Indeed, additional comparisons and analysis of our electrophysiological data indicated an effect of the *Cre^Tam^* transgene on WT *Shank3^+/+^* synaptic transmission (Fig. 9*A*), with *Cre^Tam^*+ WT mice having markedly reduced I/O curves compared to *Cre^Tam^−* WT mice (Fig. 9*A*). This was in contrast to a lack of effect of *Cre^Tam^* on mutant *Shank3^G/G^* synaptic transmission (Fig. 9*B*). The presence of the *Cre^Tam^* gene had no effect on the amplitude of the fiber volley in either *Shank3^+/+^*or *Shank3^G/G^* mice, suggesting this effect is not due to a decrease in axon number or presynaptic excitability.

## Discussion

Conditional gene targeting can allow for regulated spatial or temporal control of gene expression (Hayashi and McMahon, 2002) and has been successfully harnessed in mouse models of autism (Guy et al., 2007; Clement et al., 2012; Silva-Santos et al., 2015; Mei et al., 2016). In the ideal experiment, nuclear localization of Cre-recombinase on tamoxifen administration restores WT gene expression and rescues neuronal function and behavior. These studies can also offer insights into the critical windows in development for successful rescue (Clement et al., 2012; Silva-Santos et al., 2015; Mei et al., 2016). The potential clinical impact of adult genetic reversal is even greater, offering an avenue for permanent restoration of normal function even after brain development is complete (Van Duyne, 2015).

We sought to apply adult genetic rescue to our robust *Shank3^G^* Exon 21 mouse model of autism (Speed et al., 2015) by crossing the original *Shank3^G^* mouse line with another that expresses tamoxifen-inducible Cre recombinase under the CMV-chicken actin promotor, CAGGCre-ER (Hayashi and McMahon, 2002), abbreviated here as Cre^Tam^. This Cre^Tam^ mouse results in widespread, tamoxifen-inducible, Cre-recombinase activation. The Cre^Tam^ mouse line used in this study is well-described in the literature and has been used to rescue phenotypes in other mouse models of autism and neuropsychiatric disorders (Guy et al., 2007; Clement et al., 2012; Silva-Santos et al., 2015; Mei et al., 2016).

Our study had two objectives. First, we validated our *Shank3^G^Cre^Tam^* mouse line in *Cre^Tam^−* mice by replicating the synaptic and behavioral phenotypes identified in the original *Shank3^G^* mouse (Speed et al., 2015), and in our earlier *Shank3^ΔC^* mouse (Kouser et al., 2013). As expected, Shank3^G/G^Cre^Tam^− mice exhibited a novelty avoidance phenotype, hypoactivity in response to a novel environment, motor coordination deficits, and decreased hippocampal synaptic transmission compared to *Shank3^+/+^Cre^Tam^−* controls, faithfully replicating the strongest phenotypes observed in the original *Shank3^G^* (Speed et al., 2015) and *Shank3^ΔC^* (Kouser et al., 2013) mouse lines. This was true of both tamoxifen and vehicle-treated *Cre^Tam^−* cohorts. We chose not to examine anxiety-like behaviors or other behavioral tasks that we previously demonstrated were not affected in this mutant mouse model.

The second objective of our study was adult-induced reversal of those phenotypes in tamoxifen-treated *Shank3^G^Cre^Tam^+* mice. We achieved complete rescue of WT SHANK3 expression in adult, tamoxifen-treated *Shank3^G^Cre^Tam^+* mice using our six-week tamoxifen treatment, similar to our previous report (Speed et al., 2015). Synaptic transmission deficits also appeared to be rescued with tamoxifen treatment in *Shank3^G^Cre^Tam^+* mice, suggesting that synaptic function could be restored in adult animals. Our behavioral data were less promising with rescue of both nesting behavior and rotarod performance, but not marble burying or initial hypoactivity in the locomotor activity test.

When taken at face value before further examination of *Cre^Tam^*+, vehicle-treated controls, our data suggest conditional rescue of some *Shank3^G^* phenotypes following adult, conditional, genetic reversal. For completeness, we included a key additional control. We analyzed behavior and synaptic transmission in *Cre^Tam^+* mice treated with vehicle. We expected this to yield the same phenotypes as the original Shank3*^G^* and *Shank3^G/G^Cre^Tam^−* mice. This was true for marble burying and initial hypoactivity in a novel environment, but we still observed apparent rescue of nesting behavior, rotarod performance, and synaptic transmission even in the absence of tamoxifen. On closer inspection, we noticed that synaptic transmission in *Cre+* WT animals was reduced to *Shank3^G/G^* levels in the presence of the transgene. Thus, we are unable to conclude that adult, genetic reversal of our *Shank3* exon 21 insertion mutants is effective. Furthermore, our data suggest that *Cre^Tam^* may be having an effect on synaptic transmission and behavior, thus giving the appearance of rescue.

While we were performing our experiments, a publication from Dr. Guoping Feng’s laboratory demonstrated genetic reversal of WT SHANK3 expression, striatal physiology, and some behaviors by crossing the same *Cre^Tam^* mouse line with a different *Shank3* mutant model targeting exons 13–16 (*Shank3^PDZ^*). Tamoxifen treatment of the *Shank3^PDZ^*mutant led to rescued expression of most major protein isoforms of SHANK3 when compared to vehicle-treated *Shank3^PDZ^* mutants (Mei et al., 2016). They initially replicated behavioral and synaptic deficits in their floxed *Shank3^PDZ^* mutants without first crossing them to *Cre^Tam^*. Furthermore, this study compared *WTCre^Tam^+* and *Shank3^PDZ^Cre^Tam^+* mice treated with tamoxifen to one another, using vehicle-treated *Shank3^PDZ^Cre^Tam^+* mice as an additional comparison group (Mei et al., 2016). They did not perform a comparison between vehicle-treated *WTCre^Tam^*+ and *Shank3^PDZ^Cre^Tam^*+ mice to rule out effects of the *Cre^Tam^* transgene independent of tamoxifen treatment (Mei et al., 2016). Our data suggest that such a comparison may lead to apparent rescue of a subset of behavioral and synaptic phenotypes, at least on the *Shank3^G/G^* mutant background. They did, however, see additional, broader rescue of behaviors with tamoxifen treatment earlier in life, suggesting that these additionally rescued behaviors earlier in development were not likely due to spurious effects of *Cre^Tam^*alone in their experiments.

There are, of course, other important differences in methodology between the Mei et al. (2016) study and ours. First, our mouse model targets exon 21 of the *Shank3* gene and results in loss of all three major isoforms of SHANK3 (Speed et al., 2015) whereas the Mei et al., study targets exons 13–16 which disrupts the PDZ domain and results in loss of different isoforms of SHANK3 (Sheng and Kim, 2000; Mei et al., 2016). Tamoxifen treatment procedures also differed between studies. We administered tamoxifen in chow fed to adult mice for six weeks, while Mei et al. (2016) used an oral gavage protocol at 5–8 mg/d depending on mouse weight. Neither study observed tamoxifen toxicity in adult mice, but Mei et al. (2016) did report tamoxifen toxicity in three-week-old mice. Both groups adhered to a two-week washout period before testing.

Our adult genetic rescue experiments included a complete set of controls for a total of 12 groups tested, including controls for the *Shank3^G^* allele (heterozygous and homozygous), the *Cre^Tam^* transgene, and tamoxifen treatment. Mei et al. (2016) tested three groups in their adult genetic reversal experiments: *Shank3^+/+^Cre^Tam^+* treated with tamoxifen, homozygous *Shank3^PDZ^Cre^Tam^+* with vehicle, and homozygous *Shank3^PDZ^Cre^Tam^+* with tamoxifen, with no reported controls for tamoxifen effects or for *Cre^Tam^* transgene effects in WT mice. Furthermore, in the Mei et al. (2016) study, the homozygous mutant mice were bred separately from a cross of heterozygotes crossed with homozygotes to generate *Cre^Tam^+* homozygous mice for treatment with vehicle or tamoxifen. The *Shank3^+/+^Cre^Tam^+* mice and homozygous *Shank3^PDZ/PDZ^Cre^Tam^+* mice used in their experiments were generated from completely separate crosses of *Shank3^+/+^Cre^Tam^+/−* mice with *Shank3^+/+^Cre^Tam^+/−* mice (to yield the *Shank3^+/+^Cre^Tam^*± WT controls) and *Shank3^PDZ/PDZ^Cre^Tam^+/−* with *Shank3^PDZ/PDZ^Cre^Tam^+/−* (to yield the *Shank3^PDZ/PDZ^Cre^Tam^*± homozygous mutant mice; Mei et al., 2016). This means that the *Shank3^+/+^*comparators were generated from a completely separate cross with different parental genotypes than the *Shank3^PDZ/PDZ^Cre^Tam^+* mutants. In our study, all mice were generated from a single parental cross of *Shank3^+/G^Cre^Tam^−* mutants with *Shank3^+/G^Cre^Tam^+*. This allowed for each group tested to have its own internal *Shank3^+/+^* control from the same parents, same *Cre^Tam^+* status, and same treatment status within a littermate pair or triplet. These efforts may decrease the likelihood of misinterpretation of our adult genetic rescue experiments in this study.

Overall, we can conclude that *Shank3^G^* genetic reversal results in complete reversal of SHANK3 protein expression in the brain, a result that we have demonstrated here and in our previous publication (Speed et al., 2015). We cannot conclude, however, that this biochemical rescue leads to rescue of any behavioral or synaptic phenotypes in our mutants. In fact, it appears that the *Cre^Tam^* transgene has complex effects on WT mouse synaptic transmission in WT *Shank3* ^+/+^ mice. We can also conclude that we have successfully replicated our previous behavioral and electrophysiologic findings in two previous, similar *Shank3* mutant mouse models (Kouser et al., 2013; Speed et al., 2015). We offer this study as an important cautionary tale demonstrating that any attempt at genetic reversal must be accompanied by all appropriate controls, including careful control of parental genotypes, simultaneous *Cre^Tam^* controls, and comparisons among all vehicle and tamoxifen-treated groups for accurate interpretation of results. We cannot interpret our findings in a manner that negates previously published findings using *Cre^Tam^*in other genetic models or backgrounds. We can only stipulate that our genetic reversal data are not able to be interpreted as clearly successful genetic reversal.

## Synthesis

Reviewing Editor: Alfonso Represa, INSERM Decisions are customarily a result of the Reviewing Editor and the peer reviewers coming together and discussing their recommendations until a consensus is reached. When revisions are invited, a fact-based synthesis statement explaining their decision and outlining what is needed to prepare a revision will be listed below. The following reviewer(s) agreed to reveal their identity: NONE.

Dear authors,

Your manuscript has been evaluated by two reviewers that strongly appreciated the quality of the work and the interest of the results. They propose a few changes that I'm synthesizing below and that I recommend to take into account for acceptance:

-It would be important to add some details about housing conditions of the mice (e.g., day/night rhythm, mixed- or homogenous-genotype groups, size of the housing groups).

- Please keep the same scales for the equivalent graphs in the figures 5, 6, 7, and 8 as well as within Figure 9, to ease comparisons between the different groups of mice.

- check please for grammar, particularly in the abstract. There are some typos throughout the text that should be corrected.

- Although you have shown recombination using western blot, we think that a Southern blot would further validate their western blot-recombination results.

- In Figure 4B, you can show Tam 1 week after vehicle followed by Tam 2 and 4 weeks for a better understanding of the graph.

- We noticed that unlike in other graphs, the * is not indicated in Fig 5,6,7 A&B . Please correct this.

- Was there any change in the fibre volley vs stimulus intensity checked in Fig 5, 6, 7? If so, please include it in the manuscript.

Referees were also suggesting some additional experiments that would help providing more information on the mechanisms at the synaptic level, but after discussion we concluded that performing them will require more than two months and that they were no necessary for reaching the main conclusion. However, I'm including them below for information. It might be that you have additional data on this respect that can be included in the final manuscript.

- Could the authors check for any change in anxiety level by doing the elevated plus maze and whether it is rescued.

-Did the authors investigate whether LTP as well as synaptic transmission was rescued?

-Could the authors show whether any change in AMPA/NMDA after the rescue as the synaptic transmission is restored?

- Could the authors show whether any signaling proteins downstream of NMDARs are restored?

## Author Response

Dear Dr. Represa,

We thank the reviewers and editors for their helpful comments on our manuscript, “Apparent Genetic Rescue of Adult Shank3 Exon 21 Insertion Mutation Mice Tempered by Appropriate Control Experiments”. Please see the attached manuscript with our revisions marked by red text as well as our response to reviewer suggestions below.

-It would be important to add some details about housing conditions of the mice (e.g., day/night rhythm, mixed- or homogenous-genotype groups, size of the housing groups).

Mice were housed in a reversed, 12 hr light/dark cycle and housed in mixed genotype groups of 2-4 per cage. Details were added to the Methods section as follows:

Page 3 Lines 123-128:

“Same-sex littermates with mixed Shank3 and CreTam genotypes were housed together 2-4 per cage following weaning between postnatal days P21-P28. Mice were kept on a 12:12 light:dark cycle with experiments performed during the light cycle (6:00 AM -- 6:00 PM). Mice receiving tamoxifen treatment were housed on in the same room, but on a separate rack from mice in the control condition.”

- Please keep the same scales for the equivalent graphs in the figures 5, 6, 7, and 8 as well as within Figure 9, to ease comparisons between the different groups of mice.

We have revised these graphs as requested.

Figures 5-9:

Revised to share the same axes for comparable experiments and identical layouts.

- check please for grammar, particularly in the abstract. There are some typos throughout the text that should be corrected.

We performed a thorough check by two native English speakers and by Word spell and Grammar checks. Tenses were revised to favor past tense whenever possible to keep tense as consistent as possible throughout. If there are specific issues with grammar that are systematic, we are very open to having these pointed out. We are very happy to edit further if needed.

- Although you have shown recombination using western blot, we think that a Southern blot would further validate their western blot-recombination results.

We have added Figure 4E depicting an example Southern blot of creTam+, WT and homozygous mutant brain tissue treated with vehicle or tamoxifen. This panel is reproduced below for the reviewers' convenience.

Figure 4 (E) Example Southern blot of brain tissue in CreTam+, WT and Shank3G/G homozygous mutant mice treated with Vehicle or Tamoxifen (TAM, Rev=band expected following cre-mediated recombination of floxed mutant, Mut=band expected for floxed mutant before cre recombination, WT=band expected in WT mice without mutant allele).

- In Figure 4B, you can show Tam 1 week after vehicle followed by Tam 2 and 4 weeks for a better understanding of the graph.

The figure has been revised as requested.

Figure 4B

Reordered as vehicle, 1 week, 2 week, and 4 week TAM diet. The long, continuous blot inset above the original figure 4B was divided up by treatment group to correspond to the new order of treatment groups represented in the summary graph below.

- We noticed that unlike in other graphs, the * is not indicated in Fig 5,6,7 A&B. Please correct this.

Asterix “*” were added wherever individual points were statistically significant in post-hoc tests. Otherwise, main effects are noted in each figure consistently.

- Was there any change in the fibre volley vs stimulus intensity checked in Fig 5, 6, 7? If so, please include it in the manuscript.

We did not identify any changes in fibre volley amplitudes among groups that affect our conclusions. This has been reflected in the text as follows:

Page 12 Lines 356-357:

“Fiber volley amplitude was not affected.”

Page 19 Lines 419-421:

“Fiber volley amplitude was similar between Shank3+/+CreTam− and Shank3G/GCreTam− mice suggesting no effect on presynaptic excitability or axon number.”

Page 21 Lines 461-462:

“&, as are fiber volley amplitudes.”

Page 25 Lines 527-529:

“The presence of the CreTam gene had no effect on the amplitude of the fiber volley in either Shank3+/+ or Shank3G/G mice, suggesting a synapse-specific mechanism rather than a decrease in axon number or presynaptic excitability.”

All figures:

Saved as TIFF files at 300 DPI as stated in ENeuro's publication requirements.

We thank the reviewers for their time and consideration of our manuscript and look forward to your final decision.

Sincerely,

Craig M. Powell, M.D., Ph.D.

## References

[B1] Blundell J, Blaiss CA, Etherton MR, Espinosa F, Tabuchi K, Walz C, Bolliger MF, Sudhof TC, Powell CM (2010) Neuroligin-1 deletion results in impaired spatial memory and increased repetitive behavior. J Neurosci 30:2115–2129. 10.1523/JNEUROSCI.4517-09.2010 20147539 PMC2824441

[B2] Boccuto L, Lauri M, Sarasua SM, Skinner CD, Buccella D, Dwivedi A, Orteschi D, Collins JS, Zollino M, Visconti P, Dupont B, Tiziano D, Schroer RJ, Neri G, Stevenson RE, Gurrieri F, Schwartz CE (2013) Prevalence of SHANK3 variants in patients with different subtypes of autism spectrum disorders. Eur J Hum Genet 21:310–316. 10.1038/ejhg.2012.175 22892527 PMC3573207

[B3] Böckers TM, Mameza MG, Kreutz MR, Bockmann J, Weise C, Buck F, Richter D, Gundelfinger ED, Kreienkamp HJ (2001) Synaptic scaffolding proteins in rat brain. Ankyrin repeats of the multidomain Shank protein family interact with the cytoskeletal protein alpha-fodrin. J Biol Chem 276:40104–40112. 10.1074/jbc.M102454200 11509555

[B4] Bonaglia MC, Giorda R, Borgatti R, Felisari G, Gagliardi C, Selicorni A, Zuffardi O (2001) Disruption of the ProSAP2 gene in a t(12;22)(q24.1;q13.3) is associated with the 22q13.3 deletion syndrome. Am J Hum Genet 69:261–268. 10.1086/321293 11431708 PMC1235301

[B5] Bonaglia MC, Giorda R, Mani E, Aceti G, Anderlid BM, Baroncini A, Pramparo T, Zuffardi O (2006) Identification of a recurrent breakpoint within the SHANK3 gene in the 22q13.3 deletion syndrome. J Med Genet 43:822–828. 10.1136/jmg.2005.038604 16284256 PMC2563164

[B6] Clement JP, Aceti M, Creson TK, Ozkan ED, Shi Y, Reish NJ, Almonte AG, Miller BH, Wiltgen BJ, Miller CA, Xu X, Rumbaugh G (2012) Pathogenic SYNGAP1 mutations impair cognitive development by disrupting maturation of dendritic spine synapses. Cell 151:709–723. 10.1016/j.cell.2012.08.045 23141534 PMC3500766

[B7] Dhar SU, DEL Gaudio D, German JR, Peters SU, Ou Z, Bader PI, Berg JS, Blazo M, Brown CW, Graham BH, Grebe TA, Lalani S, Irons M, Sparagana S, Williams M, Phillips JA 3rd, Beaudet AL, Stankiewicz P, Patel A, Cheung SW, Sahoo T (2010) 22q13.3 deletion syndrome: clinical and molecular analysis using array CGH. Am J Med Genet A 152A:573–581. 10.1002/ajmg.a.33253 20186804 PMC3119894

[B8] Etherton MR, Blaiss CA, Powell CM, Sudhof TC (2009) Mouse neurexin-1alpha deletion causes correlated electrophysiological and behavioral changes consistent with cognitive impairments. Proc Natl Acad Sci USA 106:17998–18003. 10.1073/pnas.0910297106 19822762 PMC2764944

[B9] Grabrucker AM, Schmeisser MJ, Schoen M, Boeckers TM (2011) Postsynaptic ProSAP/Shank scaffolds in the cross-hair of synaptopathies. Trends Cell Biol 21:594–603. 10.1016/j.tcb.2011.07.003 21840719

[B10] Guilmatre A, Huguet G, Delorme R, Bourgeron T (2014) The emerging role of SHANK genes in neuropsychiatric disorders. Dev Neurobiol 74:113–122. 10.1002/dneu.22128 24124131

[B11] Guy J, Gan J, Selfridge J, Cobb S, Bird A (2007) Reversal of neurological defects in a mouse model of Rett syndrome. Science 315:1143–1147. 10.1126/science.1138389 17289941 PMC7610836

[B12] Hayashi S, McMahon AP (2002) Efficient recombination in diverse tissues by a tamoxifen-inducible form of Cre: a tool for temporally regulated gene activation/inactivation in the mouse. Dev Biol 244:305–318. 10.1006/dbio.2002.0597 11944939

[B13] Jaramillo TC, Speed HE, Xuan Z, Reimers JM, Liu S, Powell CM (2016) Altered striatal synaptic function and abnormal behaviour in Shank3 exon4-9 deletion mouse model of autism. Autism Res 9:350–375. 10.1002/aur.1529 26559786 PMC4857590

[B14] Jaramillo TC, Speed HE, Xuan Z, Reimers JM, Escamilla CO, Weaver TP, Liu S, Filonova I, Powell CM (2017) Novel Shank3 mutant exhibits behaviors with face validity for autism and altered striatal and hippocampal function. Autism Res 10:42–65. 27492494 10.1002/aur.1664 PMC5274551

[B15] Jiang YH, Ehlers MD (2013) Modeling autism by SHANK gene mutations in mice. Neuron 78:8–27. 10.1016/j.neuron.2013.03.016 23583105 PMC3659167

[B16] Kool MJ, VAN DE Bree JE, Bodde HE, Elgersma Y, VAN Woerden GM (2016) The molecular, temporal and region-specific requirements of the beta isoform of calcium/calmodulin-dependent protein kinase type 2 (CAMK2B) in mouse locomotion. Sci Rep 6:26989 10.1038/srep26989 27244486 PMC4886626

[B17] Kouser M, Speed HE, Dewey CM, Reimers JM, Widman AJ, Gupta N, Liu S, Jaramillo TC, Bangash M, Xiao B, Worley PF, Powell CM (2013) Loss of predominant shank3 isoforms results in hippocampus-dependent impairments in behavior and synaptic transmission. J Neurosci 33:18448–18468. 10.1523/JNEUROSCI.3017-13.2013 24259569 PMC3834052

[B18] Lim S, Naisbitt S, Yoon J, Hwang JI, Suh PG, Sheng M, Kim E (1999) Characterization of the Shank family of synaptic proteins. Multiple genes, alternative splicing, and differential expression in brain and development. J Biol Chem 274:29510–29518. 10.1074/jbc.274.41.29510 10506216

[B19] Mei Y, Monteiro P, Zhou Y, Kim JA, Gao X, Fu Z, Feng G (2016) Adult restoration of Shank3 expression rescues selective autistic-like phenotypes. Nature 530:481–484. 10.1038/nature16971 26886798 PMC4898763

[B20] Naisbitt S, Kim E, Tu JC, Xiao B, Sala C, Valtschanoff J, Weinberg RJ, Worley PF, Sheng M (1999) Shank, a novel family of postsynaptic density proteins that binds to the NMDA receptor/PSD-95/GKAP complex and cortactin. Neuron 23:569–582. 10.1016/S0896-6273(00)80809-0 10433268

[B21] Powell CM, Schoch S, Monteggia L, Barrot M, Matos MF, Feldmann N, Südhof TC, Nestler EJ (2004) The presynaptic active zone protein RIM1alpha is critical for normal learning and memory. Neuron 42:143–153. 10.1016/S0896-6273(04)00146-1 15066271 PMC3910111

[B22] Raynaud F, Janossy A, Dahl J, Bertaso F, Perroy J, Varrault A, Vidal M, Worley PF, Boeckers TM, Bockaert J, Marin P, Fagni L, Homburger V (2013) Shank3-Rich2 interaction regulates AMPA receptor recycling and synaptic long-term potentiation. J Neurosci 33:9699–9715. 10.1523/JNEUROSCI.2725-12.2013 23739967 PMC6619703

[B23] Sheng M, Kim E (2000) The Shank family of scaffold proteins. J Cell Sci 113:1851–1856. 10806096 10.1242/jcs.113.11.1851

[B24] Silva-Santos S, VAN Woerden GM, Bruinsma CF, Mientjes E, Jolfaei MA, Distel B, Kushner SA, Elgersma Y (2015) Ube3a reinstatement identifies distinct developmental windows in a murine Angelman syndrome model. J Clin Invest 125:2069–2076. 10.1172/JCI80554 25866966 PMC4463212

[B25] Speed HE, Kouser M, Xuan Z, Reimers JM, Ochoa CF, Gupta N, Liu S, Powell CM (2015) Autism-associated insertion mutation (InsG) of Shank3 exon 21 causes impaired synaptic transmission and behavioral deficits. J Neurosci 35:9648–9665. 10.1523/JNEUROSCI.3125-14.2015 26134648 PMC4571502

[B26] Uchino S, Wada H, Honda S, Nakamura Y, Ondo Y, Uchiyama T, Tsutsumi M, Suzuki E, Hirasawa T, Kohsaka S (2006) Direct interaction of post-synaptic density-95/Dlg/ZO-1 domain-containing synaptic molecule Shank3 with GluR1 alpha-amino-3-hydroxy-5-methyl-4-isoxazole propionic acid receptor. J Neurochem 97:1203–1214. 10.1111/j.1471-4159.2006.03831.x 16606358

[B27] Van Duyne GD (2015) Cre recombinase. Microbiol Spectr 3:MDNA3-0014-2014. 10.1128/microbiolspec.MDNA3-0014-2014 26104563

[B28] Verpelli C, Dvoretskova E, Vicidomini C, Rossi F, Chiappalone M, Schoen M, DI Stefano B, Mantegazza R, Broccoli V, Böckers TM, Dityatev A, Sala C (2011) Importance of Shank3 protein in regulating metabotropic glutamate receptor 5 (mGluR5) expression and signaling at synapses. J Biol Chem 286:34839–34850. 10.1074/jbc.M111.258384 21795692 PMC3186429

